# Stratified reconstruction of ancestral *Escherichia coli* diversification

**DOI:** 10.1186/s12864-019-6346-1

**Published:** 2019-12-05

**Authors:** José Maria Gonzalez-Alba, Fernando Baquero, Rafael Cantón, Juan Carlos Galán

**Affiliations:** 1 Servicio de Microbiología. Hospital Universitario Ramón y Cajal and Instituto Ramón y Cajal de Investigación Sanitaria (IRYCIS), Madrid, Spain; 2 CIBER en Epidemiología y Salud Pública (CIBERESP), Madrid, Spain; 3 Unidad de Resistencia a Antibióticos y Virulencia Bacteriana, Madrid, Spain; 4 Red Española de Investigación en Patología Infecciosa (REIPI), Madrid, Spain

**Keywords:** *Escherichia coli*, Ancient reconstruction, Phylogeny, Evolution, Phylogroups, Stratified phylogeny, Molecular polymorphism hallmark

## Abstract

**Background:**

Phylogenetic analyses of the bacterial genomes based on the simple classification in core- genes and accessory genes pools could offer an incomplete view of the evolutionary processes, of which some are still unresolved. A combined strategy based on stratified phylogeny and ancient molecular polymorphisms is proposed to infer detailed evolutionary reconstructions by using a large number of whole genomes. This strategy, based on the highest number of genomes available in public databases, was evaluated for improving knowledge of the ancient diversification of *E. coli*. This staggered evolutionary scenario was also used to investigate whether the diversification of the ancient *E. coli* lineages could be associated with particular lifestyles and adaptive strategies.

**Results:**

Phylogenetic reconstructions, exploiting 6220 available genomes in Genbank, established the *E. coli* core genome in 1023 genes, representing about 20% of the complete genome. The combined strategy using stratified phylogeny plus molecular polymorphisms inferred three ancient lineages (D, EB1A and FGB2). Lineage D was the closest to *E. coli* root. A staggered diversification could also be proposed in EB1A and FGB2 lineages and the phylogroups into these lineages. Several molecular markers suggest that each lineage had different adaptive trajectories. The analysis of gained and lost genes in the main lineages showed that functions of carbohydrates utilization (uptake of and metabolism) were gained principally in EB1A lineage, whereas loss of environmental-adaptive functions in FGB2 lineage were observed, but this lineage showed higher accumulated mutations and ancient recombination events. The population structure of *E. coli* was re-evaluated including up to 7561 new sequenced genomes, showing a more complex population structure of *E. coli,* as a new phylogroup, phylogroup I, was proposed.

**Conclusions:**

A staggered reconstruction of *E. coli* phylogeny is proposed*,* indicating evolution from three ancestral lineages to reach all main known phylogroups. New phylogroups were confirmed, suggesting an increasingly complex population structure of *E. coli*. However these new phylogroups represent < 1% of the global *E. coli* population. A few key evolutionary forces have driven the diversification of the two main *E. coli* lineages, metabolic flexibility in one of them and colonization-virulence in the other.

## Background

Since the first description of *Escherichia coli* in 1885 by Theodore Escherich*,* several generations of researchers have been fascinated by this microorganism. *E. coli* has been extensively used, from several perspectives, as a model to understand bacterial adaptability at very different external conditions [1, 2]. The huge population diversity of *E. coli* is a reflection of this adaptability. Initially, the *E. coli* population structure was based on the recognition of four well-defined phylogroups (A, B1, B2 and D) [3], but when the first whole *E. coli* genome was sequenced in 1997, a new opportunity in the comparative genomic field was perceived for this microorganism [4]. The increasing availability of a large amount of whole *E. coli* genomes allowed an unprecedented level of discrimination in phylogenetic studies, and the possibility to perform robust evolutionary reconstructions [5]. Consequently, three new phylogroups (C, E, and F) and five cryptic clades were described, progressively revealing a more complex adaptive-evolutionary scenario of the species [6]. In recent years, some of these cryptic clades have been renamed. Currently, cryptic clade V is known as *Escherichia marmotae* and *Escherichia ruysiae* is proposed for cryptic clade III.

**Fig. 1:**
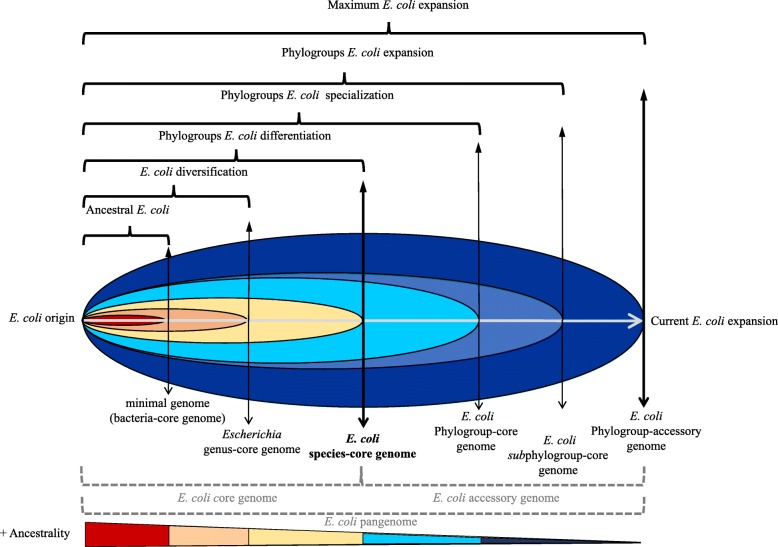
Proposal of framework for the evolutionary reconstruction of *E. coli.* The bacterial genome, classically allocated in core or accessory genome (thick vertical lines), were subdivided in order to obtain an evolutionary gradient from the most ancestral genes (core genome) to the more recently acquired (accessory genome). Different layers of analysis, reflecting the taxonomic units genetically established (bacteria, genus, species, phylogroup) and remaining genes were considered. The most ancestral set of genes corresponds to those genes identified as minimal genome (red), representing genes present in all bacteria and they probably are essential genes. *Escherichia* genus-core genome (orange) corresponds to the genes implicated in the *Escherichia* diversification prior to the formation of *E. coli* species. *E. coli* species-core genome (yellow), represents the period between the emergence of the species until the start of *E. coli* specialization*.* Phylogroup-core genome (sky blue) and *sub*phylogroups-core genome (royal blue) represents the specialization and expansion of phylogroups respectively. Phylogroup-accessory genome including the remaining genes (dark blue), to reach the current limit *E. coli* expansion

Traditionally, the evolutionary analyses of bacterial genomes have been based on the distinction of two genetic contexts, i.e., core genes pool (or core genome), including genes present in all genomes, mostly encoding basic cellular functions; and the accessory genes pool (or accessory genome), conferring strain-, pathotype- or ecotype-specific characteristics allowing adaptation to particular conditions [7]. Nonetheless, from the first available studies based on a limited number of sequences, ranging from 20 to 61 genomes [8, 9], until 250 genomes [10, 11], several discrepancies particularly regarding the origin and ancestral position of the different lineages remained unresolved. For instance, in some studies phylogroup B2 was proposed as the most ancestral *E. coli* phylogroup [2, 8, 10], whereas in other studies phylogroup D was in this ancestral position [12]. On the other hand, it was also suggested that two D separate sub-lineages could be the origin of two main evolutionary trajectories leading to lineages A/B1/C/E and B2/F [5, 13, 14]. Other researchers suggested that phylogroup B1 was the origin of E and A phylogroups [1] or proposed a paraphyletic origin for phylogroup A [15]. Other works questioned the differentiation of phylogroup C [12, 16]. We suspect that the allocation in core and accessory genomes only offers a simple, static and consequently unrealistic view of the evolutionary processes. As many genes found in bacterial genomes were acquired at different evolutionary times, our proposal was based on elucidating the successive steps in the *E. coli* diversification following the combination of two analytic approaches. On the one hand, phylogenetic reconstructions were carried out using genes sharing equivalent phylogenetic depth, thus representing the different evolutionary steps*,* from the most ancient to the most recent evolutionary ranks. These evolutionary levels successively consider the minimal genome (designed bacteria-core genome), the genus-core genome, the species-core genome, the phylogroup-core genome and the *sub*phylogroup-core genome. The remaining pool of genes, which was not assigned to these core genomes, was considered as the accessory genome (Fig. 1). This analysis, that we designated as “stratified phylogeny” (SP), permits phylogenetic reconstructions of the progressive diversification processes of *E. coli* and might identify trends that can occur over different evolutionary timescales in different lineages. We complemented the SP analysis with a second approach based on the identification of the conserved nucleotide positions among all members in each branch, but variable with respect to their hypothetical ancestor (SNPs branch-specific). This was designated as “molecular polymorphism hallmark” (mPH). This combined strategy (SP-mPH) allows the representation of the staggered diversification processes across *E. coli* lineages*.* Finally, when the ancestral reconstruction was resolved, the ancestral gains and losses of genes were studied, in order to understand the adaptive trajectories in the current lineages, and to provide some insights in the diversification drivers.

## Results

### Defining the framework for the evolutionary reconstruction of *E. coli*

At the time of starting this work, the number of available *E. coli* sequences in the genome database from NCBI was 6266 genomes. However, 46 genomes were excluded because they were wrongly classified as *E. coli* or because of very poor sequencing (Additional file 1: Figure S1). Once the remaining 6220 genomes were confirmed as *E. coli,* the classical division between core genome and accessory genome was established (Additional file 2: Table S1). *E. coli*-core genome (shared genes among all these genomes) was now defined in 1023 genes, showing a drastic reduction with respect to previous data; all remaining genes (absent in part of the population) will be automatically considered as unlimited accessory genome.

**Fig. 2:**
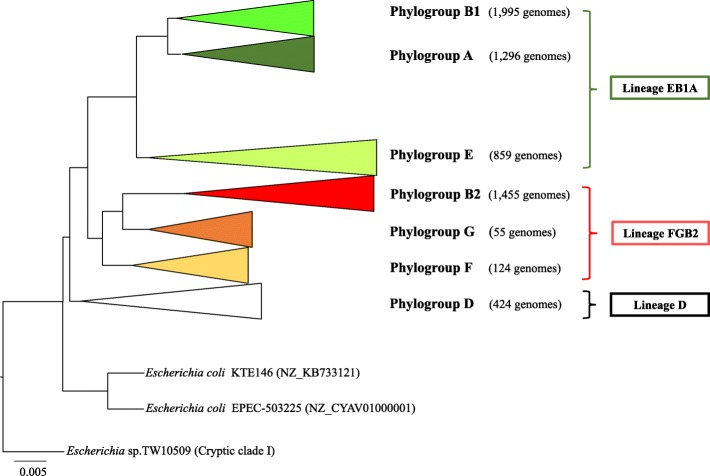
*Escherichia coli* species phylogenetic reconstruction. Phylogenetic reconstruction using Maximum-likelihood (GTR + I + Γ, SH ≥99%) with the concatenate of 1023 genes (1,040,823 nt) present in 99.9% of sequenced *E. coli* strains. All concatenate with less than 95% site coverage were eliminated. The established phylogroup C could not be distinguished in phylogroup B1. Cryptic clade I was included as outgroup in the reconstruction

A phylogenetic tree was constructed with these 1023 genes, to define the reference phylogeny, identifying the *E. coli* phylogroups. This phylogeny confirmed the previously known *E. coli* phylogroups (A, B1, B2, D, E and F), but we were unable to establish phylogroup C as monophyletic. This phylogroup came up twice independently within phylogroup B1. Among 6220 *E. coli* genomes initially studied 1296 genomes corresponded to phylogroup A (representing 20.8%), 1995 to phylogroup B1 (32.1%), 1455 to phylogroup B2 (23.4%), 424 to phylogroup D (6.8%), 859 to phylogroup E (13.8%) and 124 to phylogroup F (2%). Obviously, the number of genomes per phylogroup does not necessarily reflect the *E. coli* worldwide population (Fig. 2).

Of the studied genomes, 1.2% could not be allocated in these main phylogroups. A low diversity monophyletic clade constituted by 55 sequences (0.9% of the genomes), was located next to phylogroup B2. This phylogroup was previously proposed based on only two sequences from strains found in wild marmots [17], and tentatively designated as phylogroup G. In our study, the estimation of evolutionary divergences over sequences pairs between phylogroups confirmed the existence of phylogroup G. The remaining unclassified 10 sequences (representing 0.2% of currently known population structure) could form part of new phylogroups, but currently represent almost undetectable populations and therefore it is too early to define them as phylogroups with some degree of accuracy. Moreover, two *E. coli* genomes (KTE146 and EPEC-503225) were located in an intermediate position between the node of *Escherichia* cryptic clade I and the origin of the *E. coli* diversification. These sequences could represent ancient genomes among many bacterial lineages that have disappeared [18]. Nowadays, these sequences could be used as better candidates than cryptic clade I in the ancestral reconstruction of *E. coli* diversification, as the evolutionary distance between cryptic clade I and the *E. coli* origin is too large to consider clade I as a suitable most recent ancestor.

According to the obtained topology of *E. coli* core genome phylogeny, three main ancient lineages can be suggested. They were denominated as EB1A (including the current E, B1 and A phylogroups), FGB2 (including the current F, G, and B2 phylogroups) and D (including only phylogroup D).

### SP-mPH combined strategy yields detailed evolutionary reconstruction of the origin and diversification of *E. coli* phylogroups

Once the *E. coli* phylogeny was established in our collection, and to elucidate the first steps in the diversification and differentiation of *E. coli,* the SP-mPH strategy was applied, as detailed in the Material and Methods section. First, to examine the deepest *E. coli* in the evolutionary timescale (ancestral *E. coli* in Fig. 1), the genes previously described as belonging to the minimal genome (bacteria-core genome) [19, 20] were investigated (*n* = 206). Among all *E. coli* genomes, only 51 of these genes were found. Next, for the second level evolutionary timescale (diversification step), 189 genes corresponding to *Escherichia* genus-core genome were shared for all *Escherichia* spp., including those 51 genes of bacteria-core genome and used in the deepest stratified reconstruction (ancestral *E. coli*)*.* However, for avoiding interferences of these 51 genes used in the ancestral step, they were ignored in the new phylogenetic reconstruction (Additional file 3: Figure S2). Therefore, the remaining 138 genes were used in the diversification step. Then, in the third level evolutionary timescale (differentiation step), only those 834 genes (1023 *minus* 189 genes of the *Escherichia*-core genome) were used. A detailed list of these genes used in the different steps can be found in supplementary information (Additional file 4: Tables S2-S4). The SP-approach revealed an identical reconstruction pattern with the three evolutionary ranks (Additional file 5: Figure S3A).

Detection of single nucleotide polymorphisms (SNPs) conserved in the different phylogenetic groups (mPH-approach) could be used as high-support markers in the ancestral diversification, adding resolution to the reconstruction observed with only the SP-approach (see Material and Methods). The percentages of invariable positions were 81% among the genes belonging to ancestral *E. coli* step (corresponding to minimal genome), followed by 78% among the genes used in *E. coli* diversification step (classified as *Escherichia* genus-core) and 75% in the *E. coli* phylogroups differentiation step (*E. coli* species-core genome). The phylogroup- or lineage-specific changes present in all genomes were identified. A total number of 14 (3‰), 88 (5‰) or 490 (6‰) mutations were defined as specific in ancestral *E. coli, E. coli* diversification step, and *E. coli* phylogroup differentiation step respectively, using KTE146 and EPEC-503225 as most recent common ancestors. Subsequently, the numbers of specific changes were overprinted on the corresponding phylogenetic trees obtained using the SP-approach (Additional file 5: Figure S3B). A similar evolutionary reconstruction was obtained when cryptic clade I was used as outgroup.

**Fig. 3:**
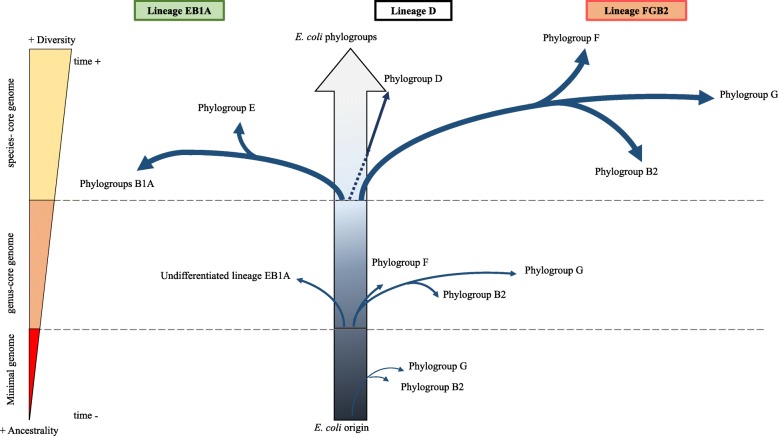
Proposed evolutionary scenario in the diversification of *E. coli* based on combined strategy (SP-mPH). The branches reflect the accumulated SNPs in conservative positions (see main text), but their lengths are not proportional to the observed evolutionary distance

SP-mPH combined strategy allowed the suggestion of a more detailed evolutionary scenario (Fig. 3) than the one obtained with core genome phylogeny. Phylogroup D, the one with fewer changes with respect to the most recent common ancestor (MRCA), was the lineage most closely related to *E. coli* origin and consequently the last phylogroup separated from the ancestral genome. SP-mPH combined strategy also allowed us to infer the successive diversification steps in EB1A and FGB2 lineages. In FGB2 lineage, we were able to identify phylogroup F as the last group separated from FGB2 root but not the first diverging phylogroup (B or G). Reconstruction of EB1A lineage only allowed us to suggest the appearance of the EB1A lineage as a non-ancient step and the subsequent separation of the AB1 sublineage.

**Fig. 4:**
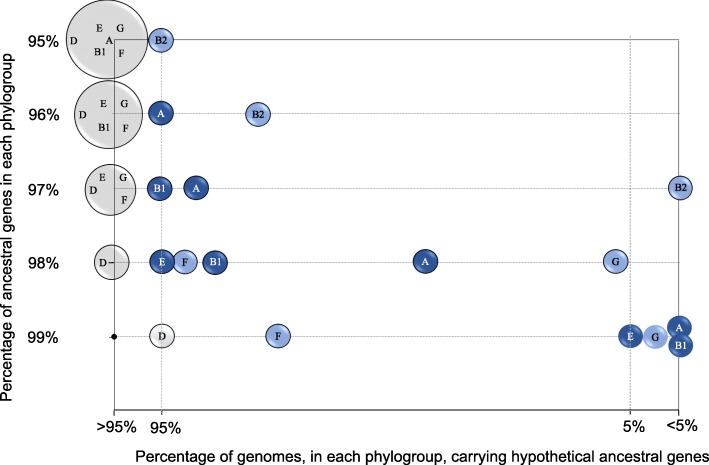
Representation of the presence of ancestral genes in each phylogroup. According to the MGRA program (http://mgra.cblab.org), the positions of the phylogroups along these horizontal lines correspond to the percentage of genomes carrying between 95 and 99% of hypothetical ancestral genes identified as ancient genome. Black point represents the hypothetical ancestral genome. Light blue circles correspond to members of FGB2 lineage and dark blue circles represent the phylogroups assigned to EB1A lineage. Lineage D is shown as a white circle. Cryptic clade I was used as outgroup in order to confirm the intermediate evolutionary position of EPEC-503225 and KTE146 strains (see main text)

To reinforce the proposed evolutionary scenario, we explored the gain and loss of ancient genes [21], reconstructing the hypothetical ancient *E. coli* core genome based on the phylogroup-core genomes, corresponding to phylogroup *E. coli* specialization in our framework (fourth level in evolutionary timescale, see Additional file 3: Figure S2). The gene content of phylogroups-core genomes ranged from 741 to 2715 genes, corresponding to phylogroups A and G respectively, once the 1023 genes corresponding to *E. coli* core genome were excluded. The analysis of the phylogroup-core genome identified a set of 2052 genes as constituting the ancient *E. coli* genome, which was then searched for in all individual genomes of each phylogroup. This analysis allowed the calculation of the percentage of genomes in each phylogroup carrying 95–99% of ancient genes. When the threshold of ancient genes was 95%, no differences among phylogroups were detectable; however, the stepwise increase of this threshold towards 99% progressively revealed differences among them (Fig. 4). Consistently with the previous analysis, phylogroup D maintained the highest percentage of strains sharing 99% of ancient genes, supporting that this was the ancestral phylogroup. Phylogroup B2 was the first in FGB2 lineage to be separated from the hypothetical ancestral genome, and phylogroup F was the last one, confirming the results obtained in the previous section. Inside the EB1A lineage, phylogroup A was the first differentiated member, while phylogroup E was the last one separated from the ancestral genome. Moreover, EPEC-503225 and KTE146 strains carried 99% of the ancestral genes, supporting our previous proposal that these strains could represent the best-to-the-present known close ancestors of *E. coli*.

Moreover, a direct correlation was found between the stratified reconstruction of *E. coli* lineages and the chromosomal sizes. For instance, in EB1A lineage, phylogroup E reached an average chromosomal size 5,364,150 bp, in phylogroup B1 it was 5,197,510 bp and phylogroup A, the first differentiated member in this lineage, had the lowest chromosomal size, with 4,977,757 bp. A similar observation was detected in FGB2 lineage. Phylogroup F showed the higher chromosomal size (average 5,321,950 bp), followed by phylogroup G with 5,245,213 bp and B2 the first separated phylogroup, yielded the lowest chromosomal size (average 5,138,164 pb) (Additional file 6: Figure S4). Significant differences in mean chromosomal sizes among phylogroups were observed (Kruskal-Wallis *p* < 0.0001), the pairwise comparisons being also significant (Mann-Whitney *p* < 0.02) except for D and G phylogroups.

### Differences in the evolutionary pathways of the major *E. coli* root lineages

To investigate if the diversification of the ancient lineages could be associated with particular lifestyles and evolutionary strategies, several genomic markers were analyzed such as the number of mutations per site, ancient recombination between and within phylogroups, and the gain or loss of genes.

The accumulated mutations per site revealed that, independently from the genes analyzed, each lineage had a distinctive mutational pattern. The less differentiated D lineage consistently showed a number of mutations below the mean value. In the EB1A lineage the accumulated mutations in recent branches were lower than mean value, whereas in the deepest branch they were higher than the mean value; On the contrary, FGB2 lineages presented the opposed pattern (Additional file 7: Figure S5). On the other hand, the analysis of ancient recombination events revealed that around 3% of the genes belonging to *E. coli* core genome had suffered recombination events. All genes identified as ancient recombinant genes were also analyzed by Gubbins program and FastGEAR program. The concordance was higher with FastGEAR (80%), but all previously identified as ancient recombinant genes were identified by one or another program. Once again, the recombination frequency was not homogeneous across ancient lineages and was more frequently found in FGB2 lineage (Additional file 8: Figure S6). The patterns of accumulated mutations per site were conserved similarly after removing the suspected recombinant genes [22]. Consequently, the three ancient lineages showed different evolutionary patterns: FGB2 yielded the highest values of accumulated mutations and recombination events, D lineage yielded the lowest mutation and recombination values, and EB1A maintained an intermediate position.

### Gain and loss of genes involved in the different adaptive processes in the main lineages

**Fig. 5:**
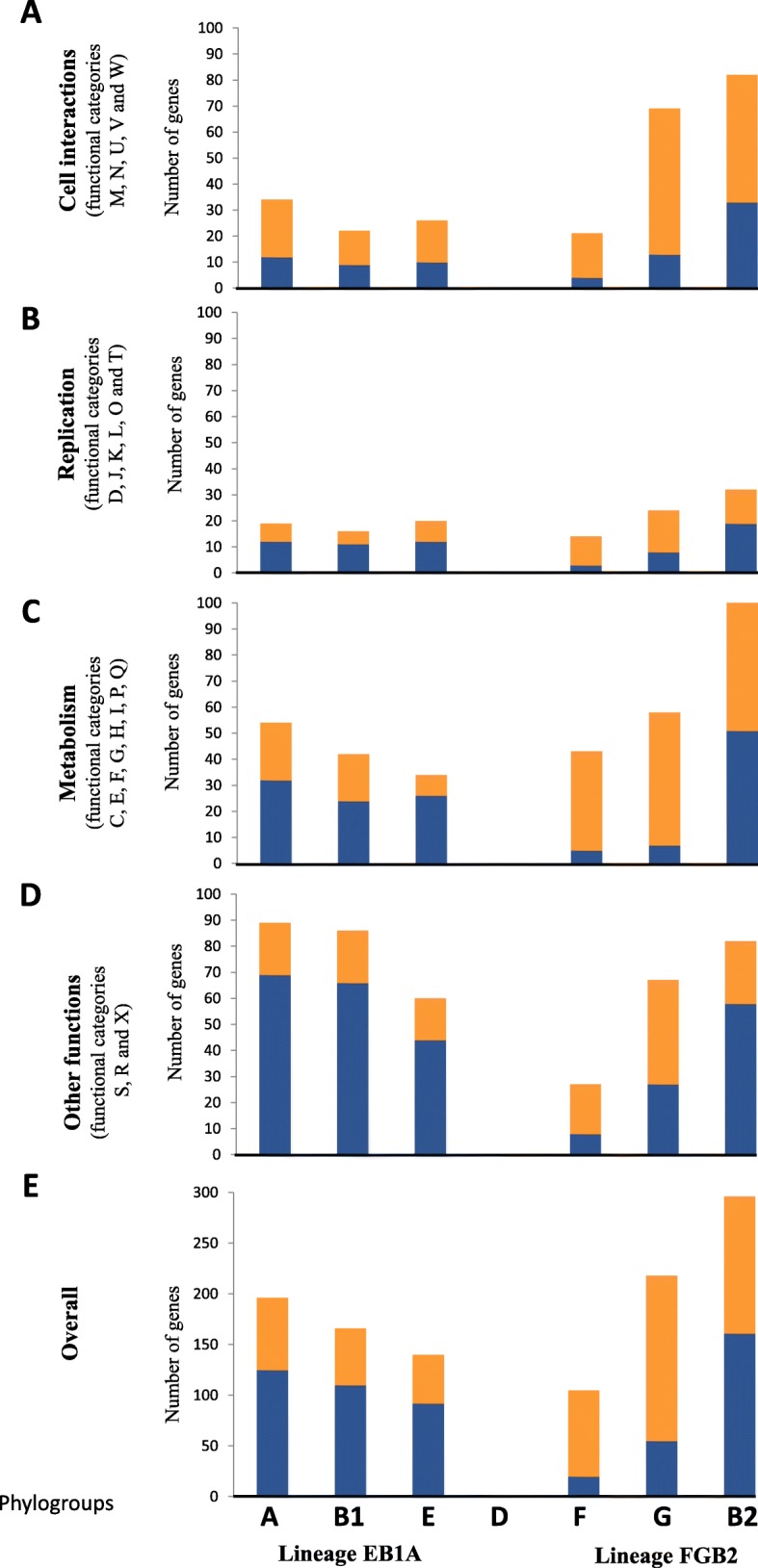
Patterns of distribution by functional categories of gained/lost gene based on Cluster Orthologous Genes (COG) classification. Four main categories were analyzed: Panel a) Cell interactions, including the functional categories M (cell wall/membrane/ envelope biogenesis), N (cell motility), U (intracellular trafficking/ secretion/ transport), V (defense mechanism) and W (extracellular structure). Panel b) Replication, including D (cell division), J (replication, ribosomal and biogenesis), K (transcription), L (replication, recombination and repair), O (post-translational modification, chaperones), T (signal transduction). Panel c) Metabolism, including C (energy production), E (amino acid transport), F (nucleotide transport), G (coenzyme transport); I (lipid transport), P (inorganic ion transport), Q (secondary metabolites). Panel d) Other functions, including S (unknown), R (general functions) and X (mobilome, prophage). Panel e) Overall representation, including all gains and losses of genes in these categories. Orange corresponds to gained genes and blue to lost genes. Phylogroup D was used as reference genome because the number of available sequences in the previously used outgroups was very low

**Fig. 6:**
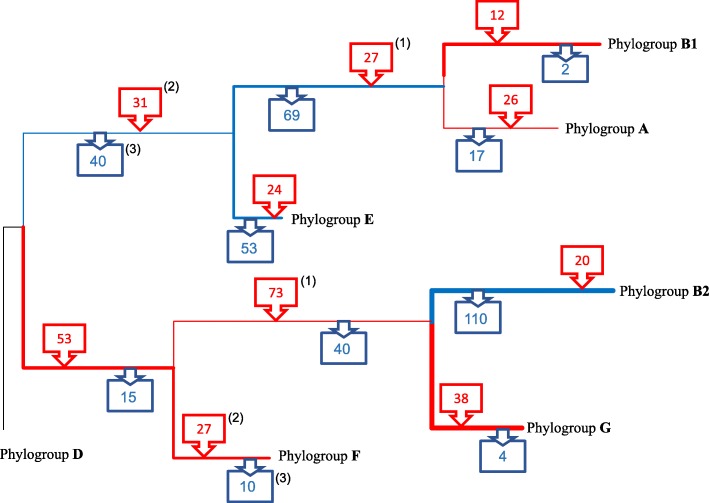
Signatures of phylogroup-core genome in the ancestral evolution of *E. coli* phylogroups. The gained/lost genes are indicated with red and blue boxes, using the phylogeny described in this work. Moreover, the thickness on the red/blue branches indicates the magnitude of gain/loss. The superscripts correspond to gained/lost genes in different trajectories indicating possible events in ecological adaptation. This representation could help to understand the different events of ecological adaptation. Phylogroup D was used as reference genome, as the number of available sequences of the *E. coli* recent ancestor was very low. ^(1)^ CreBC and bglGFBH operons; ^(2)^ dinJ-yafQ; ^(3)^ HlyD. A complete list of gained or lost genes are described in additional file 8

The gain and loss of genes was investigated searching for possible phylogroup-specific ecological adaptations. Based on the Clusters of Orthologous Groups of proteins (COGs), which classify the potential products of the studied genes in functional categories, we analysed four general categories (cell interactions, replication, metabolism, and other functions). Obviously, the three lineages should be compared with the *E. coli* ancestral genome, but this ancestral genome is no longer available (only two genomic sequences, EPEC-503225 and KTE146, could be close to the *E. coli* ancestral genome). Therefore, as the closest densely populated phylogroup to ancestral *E. coli* genome was phylogroup D, this phylogroup/lineage was used as reference. Different patterns were observed between EB1A and FGB2 lineages in these established categories. In general, FGB2 lineage gained more genes related to cell interaction, metabolism and replication than EB1A lineage (Fig. 5). Specific signatures were investigated among the members of the same phylogroup or lineage using genes according to functional annotation (Fig. 6). A complete list of gained or lost genes in each lineage, sublineage and phylogroup is described in supplementary information (Additional file 9: Table S5). Here, we only describe some examples. For instance, several independent acquisitions were identified in different branches. Among them, *creBC*, a functional two-component system involved in peptidoglycan recycling, resistance to colicins M and E2 and biofilm formation (especially in the presence of subinhibitory β-lactam concentrations) was acquired by phylogroups G and B2 in FGB2 lineage and by AB1 in EB1A lineage; Another example *is yafQ-dinJ*, a toxin-antitoxin system present in all genomes belonging to EB1A lineage and only in the F phylogroup in FGB2 lineage. These examples are rare, as the patterns of gained and lost genes are disparate among the members of the two main ancestral lineages analyzed. For instance, in the case of adhesins; if the EB1A lineage acquired the *yra* operon, the GB2 sub-lineage lost *ycg*, *ycb* and *sfm*. Similar cases were found in genes involved in the uptake of energetic nutrients.

In the EB1A lineage, the AB1 sublineage acquired genes or operons encoding enzymes related to uptake of sugars, as the *mng* operon, of the *fru*-family, involved in the uptake and metabolism of mannosyl-D-glycerate; or *xlyE*, involved in the uptake of xylose [23]. Note that a possible excess in phosphorylated sugar intermediates in cells of AB1 lineage could be detrimental, causing growth inhibition [24], because of depletion of inorganic phosphate pools. This was possibly compensated by the acquisition in the AB1 sublineage of a sugar efflux function, as the SetA transporter [25]. The *bgl* operon, encoding a phosphotransferase of the *Glc*-family system, involved in the uptake of β-glucosides, was the only phosphotransferase acquired by GB2 sublineage.

On the contrary, the AB1 sublineage lost genes encoding key proteins involved in the uptake of metals as iron, manganese and molybdene, including proteins from the siderophore metal-ABC transport system (ECSMS35_RS09855 to ECSMS35_RS09880). Genes involved in the vitamin B12 and hemin metabolism were also lost (*hmuV*, ECSMS35_RS191855 to ECSMS35_RS19215). These genes, which might influence tissue colonization and pathogenicity, were essentially preserved in phylogroup E, suggesting that AB1 sublineage could have evolved to less virulent variants compared to phylogroup E. Moreover, E phylogroup of EB1A lineage lost genes involved in the formation and processing of phosphorylated sugars such as xylulose 5-P, or ribose 5-P and ribulose 5-P. However, this phylogroup lost five genes involved in the fatty acid metabolism, suggesting deficiencies in phylogroup E for obtaining energy, if compared to AB1 sublineage.

The FGB2 lineage lost genes involved in the detoxification of benzenic aldehydes (*yag* operon or *hca* operon) [26] and genes involved in survival in extreme conditions, such as acid pH (*hyF* operon), high temperatures and low osmolarity (*yhiM*) [27]. Moreover, the phylogroup B2 lost genes with likely environmental functions, such as transport of melobiose (*melB*), utilization of cyanate as a source of nitrogen for growth (*cyn* operon), or resistance to arsenate (*ars* operon). Acquisition of toxin-antitoxin related genes was found in the FGB2 lineage. In GB2 sublineage, there was a gain of *hipA* gene, which belongs to the HipBA toxin/antitoxin system, and where the overexpression of the HipA protein leads to a persistence phenotype, including multidrug tolerance in *E. coli* [28, 29].

## Discussion

*E. coli* is the most widely sequenced microorganism, and thus provides an excellent opportunity to trace its evolutionary history in detail. Although several types of phylogenetic analyses have been published in this organism, there remain discrepant aspects concerning ancient *E. coli* phylogeny. For instance, questions on the evolutionary position of each phylogroup or the number of phylogroups that constitute the complete population structure of *E. coli* (pan-population structure) are not completely closed. Moreover, the sequencing of new genomes reveals novel combinations of traits, confirming the high plasticity and adaptive capacity of this microorganism and suggests that the *E. coli* population structure is far more complex than previously thought. In this work, a new strategy for adding resolution in the evolutionary reconstruction of bacterial species is proposed by using the largest number of *E. coli* genome assemblies available in Genbank. This strategy combines two complementary approaches. On one hand, a stratified phylogeny (SP) based on phylogenetic reconstructions with ensembles of genes representing different evolutionary timescales. On the other hand, molecular polymorphism hallmark (mPH) based on detecting conserved single nucleotide polymorphisms in the different lineages selected in different times.

The combination of SP and mPH have allowed us to propose an evolutionary scenario about the step-by-step diversification of *E. coli* species, as shown in Fig. 3. An initial limitation, found during the first stages in the development of this work, was that around 90% of the sequenced *E. coli* genomes remain in draft [30]. If draft genomes should be or not be removed from phylogenetic analysis is a matter of concern, as some genes could be lost [31]. However, the analysis of 32,000 bacterial genomes turned out the sufficient quality of the drafts for phylogenetic purposes [30].

Almost all genomes (98.8%) were allocated into previously known phylogroups. Among the extant 1.2%, the phylogroup G, that was tentatively proposed on the bases of only two genomes, was now validated, representing < 1% of the total number of sequenced *E. coli* strains. In this work, stronger evidence is presented to consider phylogroup G as a true new phylogroup. It was identified with evolutionary divergence with respect to known phylogroups which was higher than that between already established phylogroups. The core genome in phylogroup G (3,741 genes) was larger than those estimated in the previously known phylogroups (1767–2692 genes), but this result might be biased due to the low number of sequences belonging to this phylogroup. On the contrary, the proposed phylogroup H [17] (based on one single genome) still represents an ‘undetectable’ fraction in the population. Among 6220 *E. coli* genomes, we did not find any more sequences clustering in this hypothetical phylogenetic group. If phylogroup H is considered as a new phylogroup, according to Lu et al. [17], then other non-clustering sequences in an extremely low frequency could also be proposed as new phylogroups. Its apparent lack of evolutionary success does not allow us to establish it with certitude as a significant phylogroup, even when the number of available sequences was increased.

From the time this work reached its final phase at beginning of 2019, the number of whole sequenced genomes available in the GenBank database had increased to 13,781. According to the idea of a not completely known *E. coli* population structure, this wealth of information could be expected to increase the number of detectable phylogroups, describing new or resolving other cases, such as phylogroup H. We decided to build new phylogenetic trees, and to reconstruct the *E. coli* core genome again with the extended collection of genomes. Only 0.3% of these genomes were not allocated in the known phylogroups. Among them, 23 genomes (representing 0.2%) were clustered in a monophyletic branch (Additional file 10: Figure S7), which could be tentatively considered in the future as phylogroup I. (A description of the genomes identified as belonging to this new phylogroup is shown in Additional file 11: Table S6)*.* Now, the proportions of phylogroups were only slightly modified. A proportion of 35.2% genomes were classified as phylogroup B1, 24.0% as phylogroup A, 19.5% as phylogroup B2 and 10.8% as phylogroup E. The rest of the phylogroups are present in < 10% each one. Phylogroups D reached 6.5%, phylogroup F and G 2.1 and 1.6% respectively. However, we found only one genome tentatively allocated in phylogroup H. This re-analysis confirms that the *E. coli* population structure is still completely unresolved.

The *E. coli* core genome estimated with 6220 genomes was now reduced by around 322 genes with respect to the classic analysis using 61 complete genomes [9]. Although this estimation represents a significant reduction in the number of genes, this reduction maintains the core genome as 20% of the *E. coli* genome size, this data being coincidental with previous estimations [32]. The increasing availability of genomes is expected to further decrease the number of core genes. However, our estimated core genome based on 1023 genes was present in 99.9% of the 13,781 currently available genomes, so that assuming a sequencing error of 0.1% [10], it seems that we reached an estimation that could be very near the definitive *E. coli* core-genome (Additional file 12: Figure S8). Moreover, the minimal genome shared among all bacterial species should correspond to the most ancestral genetic information. Surprisingly, the number of these ancestral genes, belonging to minimal genome [19] was very low (*n* = 51), among all *E. coli* genomes. A similar approach, looking for genes of minimal genome in 63 full *E. coli* genomes, revealed that as many as 11 genes allocated as minimal genome were absent in some of these genomes [33]. That the presence of essential genes is highly variable is probably due to the phenomenon known as “non-orthologous gene displacement” which refers to the coding for an essential function by genes with no sequence homology [34].

The SP approach yielded identical reconstruction with the three evolutionary timescales, leaving questions related to ancestral stepwise diversification and differentiation processes unresolved. However, when single nucleotide polymorphism data in the mPH approach (since the conserved positions could still maintain phylogenetic information of their ancestors) was applied to understand differentiation processes at deep evolutionary timescales [35], a presumptive evolutionary scenario could be proposed. In this combined strategy, three root lineages were defined, lineage D, the deepest one, and lineages EB1A and FGB2. This evolutionary analysis suggests that early steps in the diversification of *E. coli* phylogroups started with the diversification of EB1A and FGB2 root lineages. On the other hand, the differentiation of phylogroup D only occurred much later, as this phylogroup was always located in the most basal position among the other phylogroups [5, 14], and it conserves many traits from ancestral *E. coli* genome (Fig. 4). Several groups had suggested a polyphyletic origin for phylogroup D [8, 13, 36]; however, one of these branches was now clearly identified as phylogroup F and the differentiation of phylogroup F was prior to phylogroup D (Fig. 3). Our results support other studies proposing FGB2 as the first diversified root lineage [37]. In fact, phylogroup B2 was the first differentiated phylogroup and consequently the most distant with respect to origin [2, 8, 10], losing more traits than other phylogroups from the ancestral genome. Analogous results were observed in the stepwise diversification in the EB1A lineage, where A and E phylogroups were the first and last that underwent differentiation into this lineage. In other words, phylogroups B2 and A represent the more evolved branches, whereas F and E the less evolved within their root lineages. When this evolutionary scenario is combined with the analysis of chromosomal sizes, it is very tempting to suggest a process of reductive evolution (Additional file 6: Figure S4). This has been previously proposed for phylogroup A [1], but our data suggest that this reductive process has also occurred in FGB2 and EB1A lineages.

As genomic diversification likely parallels habitat specialization, and in our case, particularly the hosts’ speciation, we tried to identify any possible signal suggesting differences in the evolutionary strategies between these ancient lineages. The differences in the frequency of mutation and recombination are indirect signals suggesting different evolutionary patterns. However, when the gain and loss of specific genes was analyzed using the COG categories, the FGB2 lineage was particularly enriched in genes involved in host’s colonization and virulence with respect to the EB1A lineage, which was more endowed (particularly the B1A sublineage) in functions assuring a more generalist style of life (see below). These results support the concept that EB1A and FGB2 lineages could be the result of the early adoption of different adaptive strategies. We investigated the acquired or eliminated specific functions at the time of differentiation of the different phylogenetic branches. Within the EB1A lineage, the B1A sublineage was enriched in carbohydrate transport systems (xylE, *bgl* operon and *mng* operon) involved in sugars uptake (xylose, aryl beta-glucosides, and mannose respectively). This might suggest a more generalist style of life, with more different available sources of energy, probably including plants, particularly in phylogroup B1 [38]. Similar results were also observed in the ancestral reconstruction of other microorganisms, such as enterococci, suggesting that the carbohydrates utilization has been the major driver of bacterial specialization [39]. However, the acquisition of the genes involved in the sugar uptake could induce a possible detrimental effect due to an excess of phosphorylated sugars [40] in the cell, and probably for this reason the B1A sublineage acquired a sugar efflux transporter (*setA*) to regulate the phosphorylated sugars concentration into the cell. On the contrary, phylogroup E lost genes involved in the metabolism of sugars (xylulose and ribulose) and fatty acid metabolism. On the other hand, phylogroup E has genes involved in the uptake of iron in the hemin metabolism (*hmuV*, ECSMS35_RS191855 to ECSMS35_RS19215), which were absent in B1A sublineage. In animals, these genes could have contributed to the pathogenesis or intestinal colonization and might represent an evolutionary convergence with FGB2 root lineage lifestyle. These results are consistent with previous findings comparing gut commensals and pathogenic *E. coli* strains [41]. Within the FGB2 root lineage, phylogroup B2, which has been suggested to be the most host-adapted, including humans [42], seems to have lost some environmental-adaptive functions. These might include those involved in transport of melobiose and cyanate, genes involved in the detoxification of benzenic aldehydes, or in the ability to grow in extreme conditions, such as acid pH high temperature, low osmolarity, or genes encoding environmentally-regulated adhesins as *ycgV*, *ycb* or *sfm*, that were consistently lost by all members of the FGB2 lineage*.* On the contrary, EB1A root lineage acquired adhesins, as *yra*, which are only expressed as response to specific environmental changes [43].

However, we cannot conclude with certainty if these ancestral events were in fact associated with adaptive processes or not. From the genes that we identified as being involved in ancestral events, most cannot be associated with known specific functions. New gene functional analysis experiments are required to sustain more solid evolutionary hypotheses. Moreover, as a consequence of high methodologic restrictions (we only consider genes present in ≥95% in a phylogroup or lineage and ≤ 5% in their ancestors), only a reduced number of genes were candidates for playing a role in ancestral processes. In the meantime, the evolutionary hypothesis presented in this work is consistent with the proposal of an early expansion of the population by acquisition metabolic flexibility advantage in the EB1A lineage and by loss of environmental-adaptive functions in FGB2 lineage [44].

This analysis includes the greatest number of available whole genomes ever used to analyze the ancestral *E. coli* diversification events, offering a new and more comprehensive view on the evolutionary history of *E. coli*. Our combined strategy of stratified phylogeny and study of molecular polymorphisms yielded a robust phylogeny, allowing for the first time a multi-level reconstruction and the indication of the possible types of genetic variation driving evolution of human adapted and environmental strains. This ancestral reconstruction between lineages can be considered as the previous, necessary step for a later reconstruction of ancestrality within lineages. This strategy can be used as a model for other detailed reconstructions of the evolutionary history of any other microorganism with a sufficient number of available sequenced genomes in databases. Recently, a similar strategy based on SNPs and gain/loss of genes was used [45]. Future research on the staggered bacterial diversification will certainly provide deeper knowledge for understanding the effect of environmental changes in microbial evolution.

## Conclusions

A new strategy for adding resolution in the evolutionary reconstruction of bacterial species is proposed. The combination of two approaches (SP-mPH strategy) allowed us to infer the step-by-step diversification of *E. coli* from ancestral lineages to current phylogroups. Moreover, this work revealed for the first time the indication of the possible types of genetic variation driving evolution of human adapted and environmental strains. The evolutionary hypothesis presented in this work is consistent with the proposal of an early expansion of the population by acquisition metabolic flexibility advantage in the EB1A lineage (more generalist style of life) and colonization and virulence in FGB2 lineage (more specialist group). On the other hand, the phylogenetic reconstruction based on > 13,000 *E. coli* sequences has allowed identification of a new phlylogroup proposed as phylogroup I, to validate the phylogroup G; moreover, phylogroup C could not be distinguished from phylogroup B1 and finally, we did not find sufficient genetic information for validating the proposed phylogroup H.

## Methods

### Data sources and selection of genes used in the different evolutionary steps

The dataset used in this work included complete and draft genome sequences of 6220 *Escherichia* species downloaded from NCBI database (https://www.ncbi.nlm.nih.gov/assembly/?term=escherichia+coli) available in August 2017. A Basic Local Alignment Search Tool (BLAST) of all-to-all genes found in the 327 complete *E. coli* genomes was performed. Protein-encoding genes with ≥70% similarity in amino acid sequence in ≥30% of sequence length were identified as homologous genes. The remaining unique genes (25,508 genes) were looked for in 6220 genomes, following the same criteria. Only those genes present in ≥99.9% of all genomes were considered as core-genome.

The genes previously identified as minimal genome (which is used here as bacteria-core genome) were searched for in all *E. coli* genomes, those identified in ≥99.9% of these genomes were used as part of *E. coli* minimal genome [19] (Additional file 4: Table S2). In a second step, those genes present in ≥99.9% available *Escherichia* genomes were defined as *Escherichia* genus-core genome (Additional file 4: Table S3). These genes were chosen and aligned using SeaView4.4 [46]. Maximum likelihood phylogeny (ML) using GTR+ I+ Γ as a model of nucleotide substitution was estimated and visualized with SeaView 4.4 program. The aLRT (approximate Likelihood Ratio Test) considered only those branches with support values > 99%. Once the operative *E. coli* genus database was established, the next steps were oriented to define the *E. coli* species-core genome, that is, the ensemble of genes present in ≥99.9% of *E. coli* genomes (Additional file 4: Table S4). Finally, phylogroup-cores genomes were established for each phylogroup.

### Framework definition

The phylogenetic reconstructions using whole genomes were based on the analysis of core and accessory genomes. The core genome was defined as the set of genes present in all members belonging to the same group (normally species). The accessory genome corresponded to the set of genes that were not present in all members of the same group. The combination of core and accessory genomes in all members of a same taxonomic unit was denominated pangenome. However, the information provided by this core-accessory allocation of genes is probably insufficient to trace evolutionary trends. Trying to overcome this limitation, we applied a combined strategy based on stratified phylogeny (SP) and molecular polymorphism hallmark (mPH) approaches The SP approach was based on stratifying the genes in four successive genomic evolutionary ranks, corresponding to the minimal (essential) genome, genus-core genome, species-core genome, and phylogroup-core genome, complemented by phylogroup-accessory genome. The hypothesis is that each one of these levels carries a different set of genes, thus providing information about the different evolutionary steps with equivalent phylogenetic depth, according to Fig. 1. The set of genes assigned to the minimal genome provides the most ancestral information (first step), as they encoded the essential functions for bacterial life, and consequently are expected to be found and evolve from the ancestral *E. coli* times. The genus-core genome included the genes present in all members of the genus *Escherichia* (second step), but excluded the genes of the minimal genome, to increase the differential features in the reconstruction of the phylogroups diversification process. *E. coli* species-core included the genes present in all members of *E. coli* but excluded the genes used in the previous steps (third step). Phylogroup-core genome included the genes present in all members of the phylogroup (“core” in phylogroup but “accessory” in *E. coli* as species) but excluded the genes present in all members of *E. coli* (fourth step). They could be candidates to help us to understand the adaptive gain/loss events between phylogroups and to reconstruct the differentiation process within phylogroup in subpopulations. In each phylogroup, new clades could be distinguished (support > 95%) and the *sub*phylogroup-core genomes were defined (fifth step). Finally, remaining genes present in a phylogroup and not included in any of the *sub*phylogroup-core genomes were classified as phylogroup-accessory genome. They could be candidates to describe the recent events and could help us to understand the adaptive possibilities of each subpopulation and probably the future sub-specialization in new subpopulations (*E. coli* expansion). The mPH approach was based on the SNPs for the reconstruction at deep evolutionary timescales [35]. First, the conserved positions in all genomes of each phylogroup were identified, and only those variable positions with respect to their hypothetical ancestor were retained. The number of selected SNPs was overprinted on the different branches in *E. coli* phylogeny previously established.

### Current and ancestral phylogeny reconstruction

The analyses with high number of sequences require high capacity of computation. To alleviate the burden of computer-time required to reconstruct large phylogenies, fast algorithms are necessary. In successive steps, phylogenies of concatenated genes were reconstructed by ML with RAxML v 8.0 (Randomized Axelerated Maximum Likelihood) [47] using GTR + I + Γ as a model of nucleotide substitution. For estimating the bootstrap values on the inferred topology by RAxML, SH test using FastTree program was used (support > 99%) [48]. To classify all genomes in their corresponding phylogroups, the following reference sequences were used for the identification of the branches, NC_000913 as phylogroup A, NC_013361 as phylogroup B1, NC_009801 as phylogroup C, NC_002655 as phylogroup E, NC_017644 as phylogroup B2, NC_010498 as phylogroup F and CU928163 as phylogroup D. New monophyletic groups with more than 10 sequences were considered as new phylogroups. The orphan sequences (lower than *n* = 10 sequences) were excluded in successive analyses. We considered as a necessary requirement to define a new phylogroup that the estimated evolutionary distance between the hypothetical new group and known phylogroups had to be higher than the distance among previously established phylogroups. Evolutionary distance between two phylogroups was obtained considering the relative length of the branches. The mean intragroup evolutionary distance was estimated as the mean distance of each branch to the origin of the phylogroup, the subtree of each phylogroup was obtained from the tree and the distances were extracted with the TreeStat program included in the BEAST software (tree.bio.ed.ac.uk/software/beast/). According to the mPH approach, the invariant positions (100% consensus sequence) present in all genomes of the same phylogroups were identified using SeaView4.4. Among the conserved positions, polymorphic sites were selected using DnaSP software [49]. These positions were used to reconstruct the evolutionary history using the parsimony method available in Mesquite program (www.mesquiteproject.org).

A second strategy based on the reconstruction of hypothetical *E. coli* ancestral core genome was implemented to reinforce the results obtained with combined strategy SP-mPH. This ancestral genome was estimated by applying the MGRA program (Multiple Genome Rearrangements and Ancestors), a tool for reconstruction of ancestral gene orders and the history of genome rearrangements (mgra.cblab.org), using phylogroup-core genome and cryptic clade I as outgroup.

### Inferring the accumulated mutation and recombination rates in each phylogroup along time

The evolutionary distances represent the accumulated mutations per site. These data provide the mean and 95% of confidence interval of the evolutionary distances of the different ancestral branches. Those branches with values of accumulated mutations higher or lower than mean value can then be distinguished. The ancient recombination in *E. coli*-core genome was suspected when the topology for individual genes tree showed inconsistency for all members belonging to the same phylogroup (ML using GTR + I + Γ as a model of nucleotide substitution) compared to the topology of the *E. coli* species-core genome tree. A limitation of this approach is the lack of support, because sometimes the phylogenetic noise is high. To avoid this limitation, the consensus phylogroup sequence for each gene (set consensus the default threshold) was defined for phylogroups. This approach reduced the noise and also excluded the non-ancient recombination. Finally, the inconsistencies were analyzed with the tree-puzzle 5.2 program [50] and SH test (*p* < 0.05). These results were re-tested using two new programs, Gubbins (Genealogies Unbiased By recomBinations In Nucleotide Sequences) available in https://sanger-pathogens.github.io/gubbins/ and FastGEAR (Fast GEnomic ARrangement) available in https://users.ics.aalto.fi/~pemartti/fastGEAR/.

### Chromosomal size for all *E. coli* phylogroups

When all genomes were allocated in phylogroups, the mean chromosomal size was calculated with confidence level 95% using SPSS program. The statistical comparison among all phylogroups was estimated using Kruskal-Wallis nonparametric tests for comparing K-independent samples or the Mann-Whitney nonparametric two-sample tests.

### Gained and lost genes between the main lineages and among different phylogroups into same lineages

This approach was applied to identify those genes segregated during early stages of diversification/specialization. For the identification of ancestral segregation, a threshold of 95–5% with respect to ancestor nodes was defined for assigning a gene as present or absent respectively. The presence/absence of genes was inferred by the asymmetric Wagner parsimony method, available in COUNT software package, (http://www.iro.umontreal.ca/~csuros/gene_content/count.html), using the *E. coli*-core genome phylogeny, quantifying the incoming and outgoing genes in consecutive ancestral nodes of the tree. If a determined gene was lost (or gained) in two phylogroups sharing a common ancestor, only a single event (loss or gain) was considered. If they did not share a common ancestor, we considered that two independent events had occurred. In this way we calculated how many genes, and how many times the studied genes in each branch of the *E. coli* tree were gained or lost.

Once the gained/lost genes were identified, they were classified based on their known or presumptive functions. The conserved domains in each gene were analyzed using CD-search tool which allowed allocation of the genes in functional COG categories (www.ncbi.nlm.nih.gov/COG). We condensed these COG-categories in four super-categories: Group A: Cell interactions, including genes presumptively involved in host-bacterial interactions, including the functional COG codes M, N, U, V and W corresponding to cell wall, membrane and envelope biogenesis (M); cell motility (N); intracellular trafficking, secretion, transport (U); defense mechanism (V); extracellular structures (W). Group B: Replication, including COG codes D, J, K, L, O and T, corresponding to cell division and chromosome partitioning (D), replication, ribosomal and biogenesis (J), transcription (K), replication, recombination and repair (L), post-translational modification, protein turnover, chaperones (O), signal traduction mechanism (T). Group C: Metabolism, including C, E, F, G, H, I, P and Q, corresponding to energy production and conversion (C), aminoacid transport and metabolism (E), nucleotide transport and metabolism (F), carbohydrate transport and metabolism (G), coenzyme transport and metabolism (H), lipid transport and metabolism (I) inorganic ion transport and metabolism (P), secondary metabolites, transport and metabolism (Q). Group D: Other functions including S (unknown), R (general function) and X (mobilome, prophages and transposons). Consistent with the aim of discovering unique properties involved in the evolutionary processes of each lineage or node of diversification, the functional characteristics of genes specifically present or absent in the phylogenetic groups were examined. (Additional file 8: Figure S6).

## Supplementary information

**Additional file 1: Figure S1.** Escherichia genus phylognetic reconstruction. (PPTX 82 kb)

**Additional file 2.** E. coli genomes examined. (CSV 1906 kb)

**Additional file 3: Figure S2.** Distribution of E. coli genes used in the evolutionary steps. (PPTX 251 kb)

**Additional file 4: Table S2.** Genes defined as core and minimal genome.

**Additional file 5: Figure S3.** Ancestor phylogenetic reconstruction of E. coli phylogroups. (PPTX 77 kb)

**Additional file 6: Figure S4.** Chromosomal size of E. coli phylogroups. (PPTX 44 kb)

**Additional file 7: Figure S5.** Inferred frequencies of accumulated mutations in the E. coli branches. (PPTX 49 kb)

**Additional file 8: Figure S6.** Ancestral recombination between E. coli phylogroups. (PPTX 59 kb)

**Additional file 9: Table S5.** Ancestral genes gain and loss by E. coli phylogroups.

**Additional file 10: Figure S7.** E. coli phylogroup I phylogeny. (PPTX 119 kb)

**Additional file 11: Table S6.** Genomes belonging to phylogroup I.

**Additional file 12: Figure S8.** Estimation of E. coli core genome. (PPTX 96 kb)

## Data Availability

All genomes are available in Genbank and the data obtained during this work are shown in main text or supplementary material.

## Abbreviations

aLRT: Approximate Likelihood Ratio Test
BEAST: Bayesian Evolutionary Analysis Sampling Trees
BLAST: Basic Local Alignment Search Tool
COGs: Clusters of Orthologous Groups
GTR: General Time Reverse
MGRA: Multiple Genome Rearrangements and Ancestors
ML: Maximum likelihood
mPH: Molecular Polymorphism Hallmark
MRCA: Most Recent Common Ancestor
RAxML: Randomized Axelerated Maximum Likelihood
SH: Shimodaira-Hasegawa
SNPs: Single Nucleotide Polymorphisms
SP: Stratified Phylogeny
