# Supplementary material for: Stratified reconstruction of ancestral Escherichia coli diversification
Source: BMC Genomics. 2019 Dec 5;20:936. doi: 10.1186/s12864-019-6346-1 (PMC6896753; doi:10.1186/s12864-019-6346-1)
Supplement: Supplementary file 1 — Additional file 1: Figure S1. Escherichia genus phylognetic reconstruction. (PPTX 82 kb) [file 12864_2019_6346_MOESM1_ESM.pptx]

## Slide 1
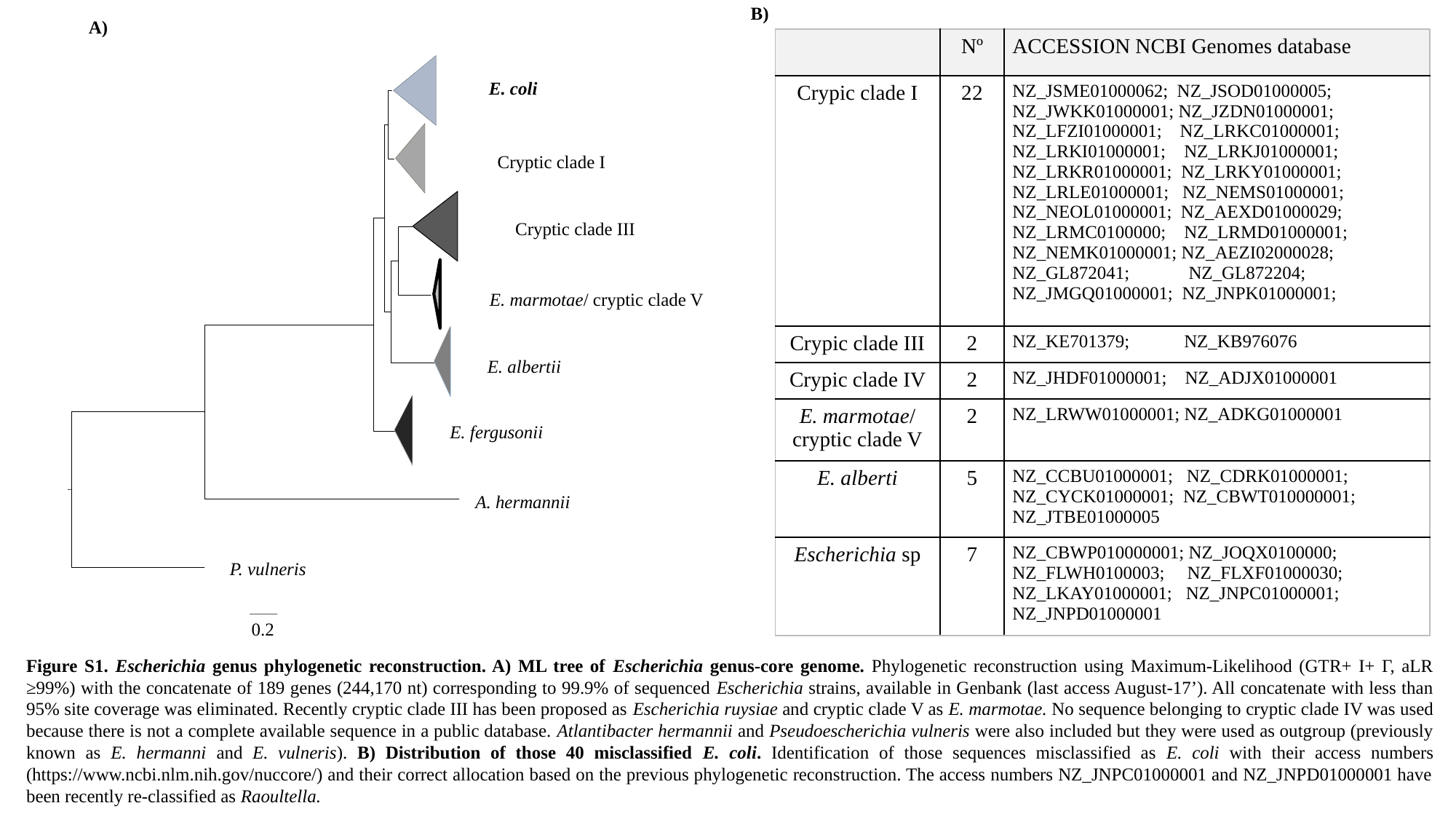

B)
A)
| | Nº | ACCESSION NCBI Genomes database |
| --- | --- | --- |
| Crypic clade I | 22 | NZ\_JSME01000062; NZ\_JSOD01000005; NZ\_JWKK01000001; NZ\_JZDN01000001; NZ\_LFZI01000001; NZ\_LRKC01000001; NZ\_LRKI01000001; NZ\_LRKJ01000001; NZ\_LRKR01000001; NZ\_LRKY01000001; NZ\_LRLE01000001; NZ\_NEMS01000001; NZ\_NEOL01000001; NZ\_AEXD01000029; NZ\_LRMC0100000; NZ\_LRMD01000001; NZ\_NEMK01000001; NZ\_AEZI02000028; NZ\_GL872041; NZ\_GL872204; NZ\_JMGQ01000001; NZ\_JNPK01000001; |
| Crypic clade III | 2 | NZ\_KE701379; NZ\_KB976076 |
| Crypic clade IV | 2 | NZ\_JHDF01000001; NZ\_ADJX01000001 |
| E. marmotae/ cryptic clade V | 2 | NZ\_LRWW01000001; NZ\_ADKG01000001 |
| E. alberti | 5 | NZ\_CCBU01000001; NZ\_CDRK01000001; NZ\_CYCK01000001; NZ\_CBWT010000001; NZ\_JTBE01000005 |
| Escherichia sp | 7 | NZ\_CBWP010000001; NZ\_JOQX0100000; NZ\_FLWH0100003; NZ\_FLXF01000030; NZ\_LKAY01000001; NZ\_JNPC01000001; NZ\_JNPD01000001 |
E. coli
0.2
Cryptic clade I
Cryptic clade III
E. marmotae/ cryptic clade V
E. albertii
E. fergusonii
A. hermannii
P. vulneris
Figure S1. Escherichia genus phylogenetic reconstruction. A) ML tree of Escherichia genus-core genome. Phylogenetic reconstruction using Maximum-Likelihood (GTR+ I+ Γ, aLR ≥99%) with the concatenate of 189 genes (244,170 nt) corresponding to 99.9% of sequenced Escherichia strains, available in Genbank (last access August-17’). All concatenate with less than 95% site coverage was eliminated. Recently cryptic clade III has been proposed as Escherichia ruysiae and cryptic clade V as E. marmotae. No sequence belonging to cryptic clade IV was used because there is not a complete available sequence in a public database. Atlantibacter hermannii and Pseudoescherichia vulneris were also included but they were used as outgroup (previously known as E. hermanni and E. vulneris). B) Distribution of those 40 misclassified E. coli. Identification of those sequences misclassified as E. coli with their access numbers (https://www.ncbi.nlm.nih.gov/nuccore/) and their correct allocation based on the previous phylogenetic reconstruction. The access numbers NZ_JNPC01000001 and NZ_JNPD01000001 have been recently re-classified as Raoultella.
