# Supplementary material for: Stratified reconstruction of ancestral Escherichia coli diversification
Source: BMC Genomics. 2019 Dec 5;20:936. doi: 10.1186/s12864-019-6346-1 (PMC6896753; doi:10.1186/s12864-019-6346-1)
Supplement: Supplementary file 3 — Additional file 3: Figure S2. Distribution of E. coli genes used in the evolutionary steps. (PPTX 251 kb) [file 12864_2019_6346_MOESM3_ESM.pptx]

## Slide 1
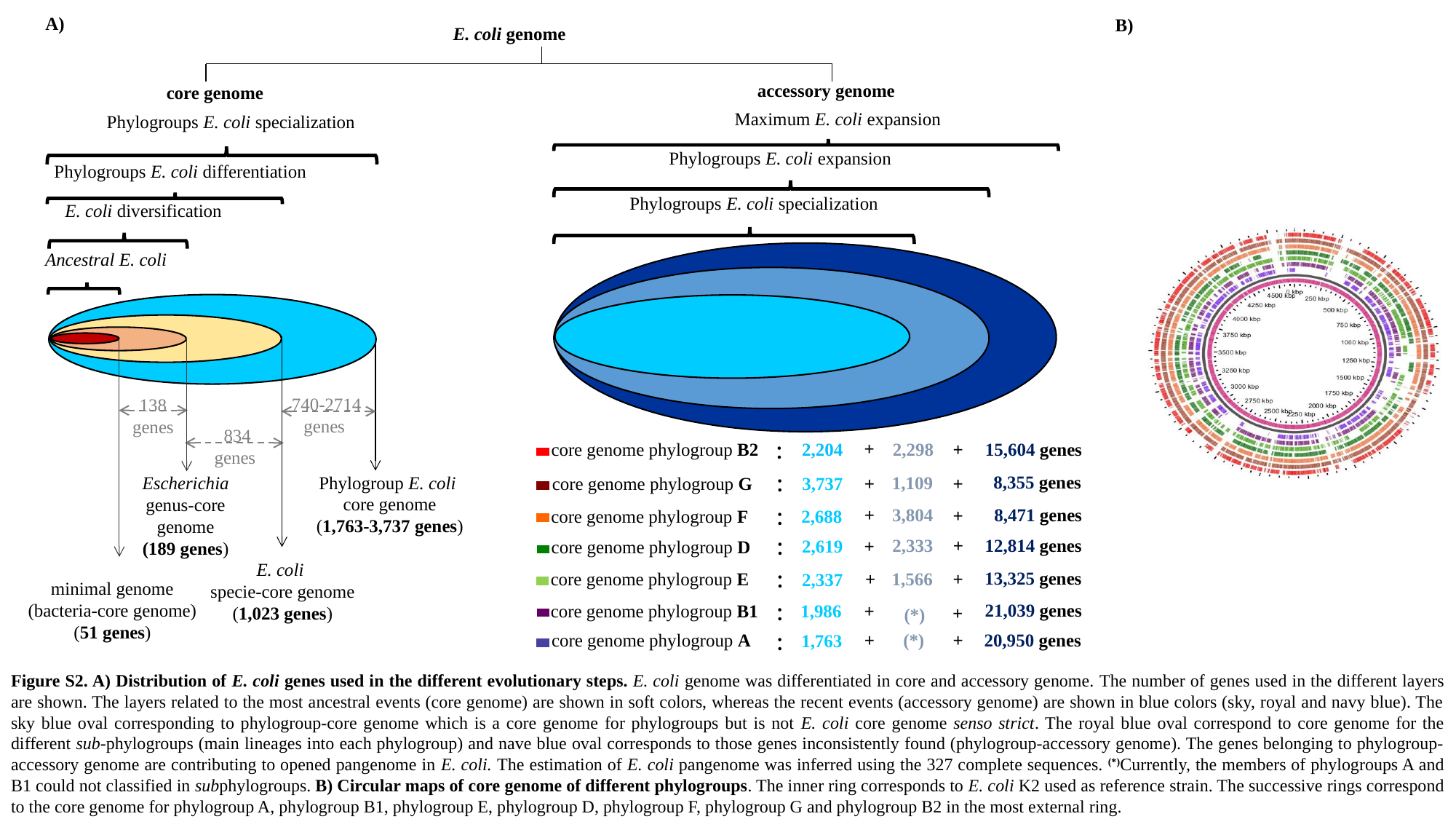

A)
B)
E. coli genome
accessory genome
core genome
Maximum E. coli expansion
Phylogroups E. coli specialization
Phylogroups E. coli differentiation
E. coli diversification
Ancestral E. coli
740-2714 genes
138
genes
834
genes
Phylogroup E. coli
core genome
(1,763-3,737 genes)
Escherichia
genus-core genome
(189 genes)
E. coli
specie-core genome
(1,023 genes)
minimal genome
(bacteria-core genome)
(51 genes)
Phylogroups E. coli expansion
Phylogroups E. coli specialization
:
+
15,604 genes
+
 2,298
core genome phylogroup B2
 2,204
8,355 genes
 1,109
3,737
core genome phylogroup G
+
+
+
8,471 genes
 3,804
+
2,688
core genome phylogroup F
 2,333
12,814 genes
+
2,619
+
core genome phylogroup D
13,325 genes
1,566
core genome phylogroup E
+
+
2,337
21,039 genes
core genome phylogroup B1
+
1,986
+
+
+
20,950 genes
core genome phylogroup A
1,763
:
:
:
:
:
(*)
:
(*)
Figure S2. A) Distribution of E. coli genes used in the different evolutionary steps. E. coli genome was differentiated in core and accessory genome. The number of genes used in the different layers are shown. The layers related to the most ancestral events (core genome) are shown in soft colors, whereas the recent events (accessory genome) are shown in blue colors (sky, royal and navy blue). The sky blue oval corresponding to phylogroup-core genome which is a core genome for phylogroups but is not E. coli core genome senso strict. The royal blue oval correspond to core genome for the different sub-phylogroups (main lineages into each phylogroup) and nave blue oval corresponds to those genes inconsistently found (phylogroup-accessory genome). The genes belonging to phylogroup-accessory genome are contributing to opened pangenome in E. coli. The estimation of E. coli pangenome was inferred using the 327 complete sequences. (*)Currently, the members of phylogroups A and B1 could not classified in subphylogroups. B) Circular maps of core genome of different phylogroups. The inner ring corresponds to E. coli K2 used as reference strain. The successive rings correspond to the core genome for phylogroup A, phylogroup B1, phylogroup E, phylogroup D, phylogroup F, phylogroup G and phylogroup B2 in the most external ring.
