# Supplementary material for: Stratified reconstruction of ancestral Escherichia coli diversification
Source: BMC Genomics. 2019 Dec 5;20:936. doi: 10.1186/s12864-019-6346-1 (PMC6896753; doi:10.1186/s12864-019-6346-1)
Supplement: Supplementary file 4 — Additional file 4: Table S2. Genes defined as core and minimal genome. [file 12864_2019_6346_MOESM4_ESM.docx]

**Table S2.** **Genes defined as minimal bacterial genome identified in *E. coli* (51 genes).** Genes present in 99.9% of 6,290 *Escherichia* genomes available in our database and previously estimated as minimal bacterial genome. The genes are identified by their name.

| atpB | plsB |
| --- | --- |
| atpC | rnpA |
| atpD | rplE |
| atpE | rplM |
| atpF | rplN |
| atpG | rplQ |
| atpH | rplT |
| cysS | rplX |
| dfp | rpmG |
| ftsY | rpmH |
| gltX | rpmI |
| glyS | rpoA |
| gmk | rpsD |
| gpsA | rpsI |
| gyrA | rpsK |
| gyrB | rpsM |
| hflB | rpsN |
| hupA | secY |
| infC | ssb |
| leuS | thrS |
| lysS | tktA |
| metK | tpiA |
| pfkA | trpS |
| pgi | tyrS |
| pgk | yidC |
| pheT |  |

**Table S3**. **Genes identified as *Escherichia* genus-core genome.** Genes present in 99.9% of 6,290 *Escherichia* genomes available in our database. The genes are identified by their name.

| accC | gltB | pck | sucB | gyrA |
| --- | --- | --- | --- | --- |
| ackA | gltD | pepN | sucC | gyrB |
| alr | glyQ | pflA | sucD | hflB |
| arcA | gntY | pflB | tdcE | hupA |
| argS | gpmI | pgm | thrA | infC |
| artP | hemN | ppiB | tldD | leuS |
| artQ | hisP | pps | trmE | lysS |
| asd | hslV | prfB | truA | metK |
| atpA | hupB | prfC | tsx | pfkA |
| bipA | ihfA | proY | uvrA | pgi |
| brnQ | ilvC | pstA | yajC | pgk |
| clpA | ilvD | pstB | ybeB | pheT |
| cpxP | ispB | pstC | ybeX | plsB |
| cpxR | livG | pstS | ybeZ | rnpA |
| crp | livH | pta | ybiT | rplE |
| cstA | livM | purF | ycaO | rplM |
| cydA | lrp | pykF | yccW | rplN |
| deoD | lysP | pyrD | yecO | rplQ |
| dnaA | malP | radA | yfiD | rplT |
| dnaB | mdh | rbsC | yhgF | rplX |
| entB | mdtB | rdgC | yiiQ | rpmG |
| fabB | mdtC | recF | yjjK | rpmH |
| fbaA | metA | rplY | yjjY | rpmI |
| fdhE | metG | rpmJ | yqiA | rpoA |
| fdnG | metH | rpoH | atpB | rpsD |
| fdoG | mglB | rpoZ | atpC | rpsI |
| fieF | mnmC | rpsA | atpD | rpsK |
| focA | mrcA | rsmC | atpE | rpsM |
| folD | mukB | sdhA | atpF | rpsN |
| gcvP | mukF | secB | atpG | secY |
| gcvT | nirB | secD | atpH | ssb |
| gidA | nrdA | secF | cysS | thrS |
| glgB | nrdB | secG | dfp | tktA |
| glmS | nuoC | sodA | ftsY | tpiA |
| glnA | ompC | speA | gltX | trpS |
| glnH | ompR | spoT | glyS | tyrS |
| glpD | parC | sspA | gmk | yidC |
| glpK | parE | sucA | gpsA |  |

**Table S4. Genes identified as *Escherichia coli* species core genome**. Genes present in 99.9% of 6,220 *E. coli* genomes available in our database. The genes are identified by their name or locus_tag identifier. ECNA114 corresponds to the sequence NC_017644, ECSMS35 corresponds to the sequence NC_010498, ECUMN corresponds to the sequence CU928163 and Z corresponds to the sequence NC_002655

| aaeA | | artP | | cpxA | | ECSMS35_RS02095 | | fdoI | | glnG | | helD | | lasT | |
| --- | --- | --- | --- | --- | --- | --- | --- | --- | --- | --- | --- | --- | --- | --- | --- |
| aaeB | | artQ | | cpxP | | ECSMS35_RS03625 | | feoA | | glnH | | hemH | | leuS | |
| aaeR | | asd | | cpxR | | ECSMS35_RS03650 | | feoB | | glnK | | hemN | | lexA | |
| aaeX | | asnA | | crcB | | ECSMS35_RS03995 | | fepA | | glnL | | hflB | | lgt | |
| aas | | asnC | | creA | | ECSMS35_RS04145 | | fepB | | glnP | | hisJ | | lhr | |
| aat | | aspC | | creB | | ECSMS35_RS04190 | | fepC | | glnQ | | hisM | | ligB | |
| accC | | aspS | | creC | | ECSMS35_RS04580 | | fepD | | glnS | | hisP | | livF | |
| aceA | | astA | | creD | | ECSMS35_RS04585 | | fepE | | gloA | | hisQ | | livG | |
| aceB | | astB | | crp | | ECSMS35_RS07340 | | fepG | | glpA | | hofM | | livH | |
| aceK | | astC | | cspD | | ECSMS35_RS10885 | | fes | | glpC | | hofN | | livJ | |
| ackA | | astD | | cstA | | ECSMS35_RS11590 | | fic | | glpD | | hofO | | livK | |
| acpT | | astE | | cueR | | ECSMS35_RS11665 | | fieF | | glpE | | hofP | | livM | |
| acrA | | atpA | | cutC | | ECSMS35_RS11695 | | fiu | | glpG | | hofQ | | lldD | |
| acrB | | atpB | | cydA | | ECSMS35_RS12115 | | fldA | | glpK | | holA | | lldP | |
| acrR | | atpC | | cydC | | ECSMS35_RS12430 | | fldB | | glpQ | | holD | | lldR | |
| ada | | atpD | | cydD | | ECSMS35_RS14060 | | focA | | glpR | | hslO | | lnt | |
| adk | | atpE | | cysE | | ECSMS35_RS16630 | | folB | | glpT | | hslR | | lolA | |
| adrA | | atpF | | cysS | | ECSMS35_RS16900 | | folD | | glpX | | hslU | | lplA | |
| aer | | atpG | | cytR | | ECSMS35_RS17515 | | folE | | gltA | | hslV | | lptA | |
| aes | | atpH | | dam | | ECSMS35_RS18585 | | folP | | gltB | | hspQ | | lptB | |
| alkB | | atpI | | damX | | ECSMS35_RS19630 | | folX | | gltD | | htpG | | lptC | |
| alr | | bacA | | dcuC | | ECSMS35_RS21385 | | fpr | | gltI | | htrG | | lpxH | |
| amiC | | baeR | | dedA | | ECSMS35_RS21390 | | fruA | | gltJ | | hupA | | lpxK | |
| amiD | | baeS | | degQ | | ECSMS35_RS21415 | | fruK | | gltK | | hupB | | lrhA | |
| amtB | | bcr | | degS | | ECSMS35_RS21435 | | fsaA | | gltL | | hyaA | | lrp | |
| anmK | | bglA | | deoB | | ECSMS35_RS24720 | | ftnA | | gltS | | hyaC | | ltaE | |
| ansA | | bglX | | deoC | | ECSMS35_RS24790 | | ftsE | | gltX | | hyaD | | lysA | |
| ansB | | bioA | | deoD | | ECUMN_3502 | | ftsK | | glyQ | | hyaE | | lysC | |
| appB | | bioB | | dfp | | elbB | | ftsN | | glyS | | hyaF | | lysP | |
| apt | | bioC | | dgkA | | emrD | | ftsX | | gmk | | iaaA | | lysR | |
| aqpZ | | bioD | | dinF | | engB | | ftsY | | gntK | | iclR | | lysS | |
| araJ | | bioF | | dinG | | entA | | fur | | gntR | | idi | | lysU | |
| arcA | | bioH | | dkgA | | entB | | galP | | gntT | | ihfA | | macA | |
| arcB | | bipA | | dmsA | | entC | | galR | | gntU | | ihfB | | macB | |
| argA | | brnQ | | dmsB | | entD | | galS | | gntX | | ilvA | | mak | |
| argD | | btuC | | dmsC | | entE | | gcvH | | gntY | | ilvC | | malP | |
| argG | | cca | | dnaA | | entF | | gcvP | | gph | | ilvD | | malQ | |
| argO | | ccmF | | dnaB | | entS | | gcvT | | gpmI | | ilvE | | malT | |
| argP | | ccmH | | dnaG | | envC | | gidA | | gpsA | | ilvY | | malZ | |
| argS | | cdh | | dnaN | | envZ | | gidB | | greB | | inaA | | mdaB | |
| argT | | cedA | | dnaX | | epd | | glgA | | grxC | | infC | | mdh | |
| aroA | | chbC | | dpiA | | exbB | | glgB | | gshB | | ispB | | mdlB | |
| aroB | | cirA | | dpiB | | exbD | | glgC | | gsiA | | katE | | mdtB | |
| aroC | | clpA | | dsbC | | fabB | | glgP | | gsiB | | kbl | | mdtC | |
| aroH | | clpS | | dusC | | fbaA | | glgX | | gsk | | kdgT | | mdtD | |
| aroK | | cmk | | dut | | fdhE | | glmM | | gst | | kdsB | | mdtQ | |
| aroL | | coaD | | ECNA114_RS03770 | | fdnG | | glmS | | gyrA | | kdsC | | menA | |
| aroM | | cobC | | ECNA114_RS15430 | | fdnH | | glmU | | gyrB | | kdsD | | menB | |
| artI | | corA | | eco | | fdoG | | glnA | | hcp | | kdtA | | menD | |
| artM | | cpdA | | ECSMS35_RS00025 | | fdoH | | glnE | | hcr | | kefA | | menE | |
| menF | | nrdA | | ppdA | | recQ | | rtcR | | sufE | | uvrB | | ybiJ | |
| mepA | | nrdB | | ppdB | | recR | | rtn | | sufI | | uvrD | | ybiN | |
| metA | | nrfE | | ppdC | | rfaD | | rumB | | sulA | | wecH | | ybiO | |
| metC | | nudE | | ppiA | | rfaE | | ruvA | | tap | | xerD | | ybiP | |
| metG | | nudF | | ppiB | | rfaF | | ruvB | | tas | | xthA | | ybiR | |
| metH | | nudG | | pps | | rhlE | | ruvC | | tdcE | | yaiA | | ybiS | |
| metK | | nuoA | | pqiA | | rhtA | | sanA | | tdh | | yaiE | | ybiT | |
| mglA | | nuoB | | pqiB | | rhtB | | sbcC | | tesB | | yaiI | | ybiV | |
| mglB | | nuoC | | prfB | | rhtC | | sbcD | | tgt | | yajB | | ybiW | |
| mgsA | | ompC | | prfC | | rihA | | sbp | | thiD | | yajC | | ybiX | |
| mnmC | | ompF | | priA | | rimI | | scpC | | thiM | | yajD | | ybiY | |
| mntR | | ompR | | priC | | rlmL | | sdhA | | thrA | | yajI | | ybjD | |
| moaA | | ompX | | prkB | | rlpA | | sdhB | | thrC | | ybaA | | ybjE | |
| moaB | | osmE | | prmB | | rlpB | | sdhC | | thrS | | ybaB | | ybjO | |
| moaC | | pabA | | proC | | rng | | sdhD | | thyA | | ybaN | | ybjP | |
| moaE | | parC | | proY | | rnpA | | secB | | tktA | | ybaY | | ybjQ | |
| moeA | | parE | | psiF | | rnt | | secD | | tldD | | ybaZ | | ybjS | |
| moeB | | pbpG | | pspG | | rob | | secF | | tolC | | ybcF | | ybjT | |
| mqo | | pck | | pstA | | rpe | | secG | | topB | | ybcI | | ybjX | |
| mrcA | | pdxB | | pstB | | rph | | secY | | torZ | | ybcJ | | ycaI | |
| mrp | | pdxH | | pstC | | rpiA | | selD | | tpiA | | ybdD | | ycaJ | |
| msbA | | pdxY | | pstS | | rplE | | seqA | | trkD | | ybdH | | ycaL | |
| mtgA | | pepE | | pta | | rplM | | serA | | trmE | | ybdZ | | ycaO | |
| mukB | | pepN | | ptr | | rplN | | serB | | trmH | | ybeB | | ycaP | |
| mukF | | pepP | | ptsN | | rplQ | | serC | | trpR | | ybeL | | ycaQ | |
| murA | | pfkA | | ptsP | | rplT | | sfsB | | trpS | | ybeM | | ycaR | |
| mutH | | pfkB | | purE | | rplX | | siiDA | | truA | | ybeX | | ycbB | |
| mutM | | pflA | | purF | | rplY | | slmA | | trxB | | ybeY | | ycbC | |
| nadD | | pflB | | purK | | rpmG | | slt | | tsgA | | ybeZ | | ycbJ | |
| nadE | | pgi | | purR | | rpmH | | slyA | | tsx | | ybfM | | ycbK | |
| nadR | | pgk | | pykF | | rpmI | | smtA | | tyrB | | ybfN | | ycbL | |
| nagA | | pgm | | pyrD | | rpmJ | | sodA | | tyrP | | ybgH | | ycbW | |
| nagB | | pheS | | pyrE | | rpoA | | speA | | tyrS | | ybgI | | ycbX | |
| nagC | | pheT | | qor | | rpoD | | speB | | ubiA | | ybgK | | yccA | |
| nagD | | phoA | | qseB | | rpoH | | spoT | | ubiC | | ybgL | | yccF | |
| nagE | | phoB | | qseC | | rpoN | | sppA | | ubiG | | ybhB | | yccK | |
| napA | | phoR | | queA | | rpoZ | | spr | | ubiH | | ybhF | | yccR | |
| napD | | phoU | | radA | | rppH | | sprT | | ubiX | | ybhG | | yccS | |
| napF | | pitA | | radC | | rpsA | | spy | | ugpA | | ybhK | | yccT | |
| nei | | pldA | | rarD | | rpsD | | ssb | | ugpC | | ybhL | | yccU | |
| nemA | | pldB | | rbsA | | rpsI | | sspA | | ugpE | | ybhN | | yccW | |
| nfo | | plsB | | rbsC | | rpsK | | sspB | | ugpQ | | ybhO | | ydgA | |
| nikA | | plsC | | rbsD | | rpsM | | ssuB | | uhpA | | ybhP | | ydhA | |
| nikB | | pncA | | rcsC | | rpsN | | ssuC | | uhpB | | ybhQ | | ydhB | |
| nikC | | pncB | | rdgC | | rpsU | | ssuD | | uhpC | | ybhR | | ydhC | |
| nikD | | polA | | recB | | rsmB | | sucA | | uhpT | | ybhS | | ydhD | |
| nikE | | potG | | recC | | rsmC | | sucB | | uidC | | ybiB | | ydhM | |
| nikR | | potH | | recD | | rsmD | | sucC | | usg | | ybiC | | ydhO | |
| nirB | | potI | | recF | | rsuA | | sucD | | uup | | ybiH | | ydhP | |
| npr | | poxB | | recJ | | rtcA | | sufD | | uvrA | | ybiI | | ydhV | |
| ydiA | | yehW | | yfbR | | yggR | | yheU | | yidB | | yjiY | | yqgE | |
| ydiE | | yehX | | yfbS | | yggS | | yhfA | | yidC | | yjjG | | yqgF | |
| ydiH | | yehY | | yfbT | | yggT | | yhfG | | yidF | | yjjI | | yqhC | |
| ydiI | | yehZ | | yfbU | | yggU | | yhfK | | yidG | | yjjK | | yqiA | |
| ydiJ | | yeiB | | yfbV | | yggV | | yhgA | | yidH | | yjjW | | yqiB | |
| ydiK | | yeiE | | yfcA | | yggW | | yhgE | | yieM | | yjjX | | yraM | |
| ydiU | | yeiG | | yfcC | | yghB | | yhgF | | yieN | | yjjY | | yraN | |
| ydiY | | yeiH | | yfcH | | ygiA | | yhgG | | yigB | | ylaB | | yrbA | |
| ydjA | | yeiI | | yfcL | | ygiB | | yhgN | | yigI | | yliI | | yrbB | |
| ydjM | | yeiJ | | yfcM | | ygiC | | yhhA | | yigL | | yliK | | yrbC | |
| ydjN | | yeiS | | yfcN | | ygiE | | yhhK | | yihI | | ynhG | | yrbD | |
| ydjQ | | yeiT | | yfiD | | ygiF | | yhhL | | yihX | | yniC | | yrbE | |
| ydjR | | yeiU | | ygdB | | ygiH | | yhhM | | yiiD | | ynjA | | yrbF | |
| ydjZ | | yejA | | ygdQ | | ygiN | | yhhN | | yiiM | | ynjB | | yrbG | |
| yebB | | yejB | | ygdR | | ygiP | | yhhW | | yiiQ | | ynjC | | yrbL | |
| yebC | | yejE | | ygeA | | ygiV | | yhhX | | yiiR | | ynjE | | yrfF | |
| yecA | | yejF | | ygeD | | ygiW | | yhiP | | yiiS | | ynjF | | yrfG | |
| yecD | | yejG | | ygfA | | ygjD | | yiaA | | yiiT | | ynjH | | ysaB | |
| yecE | | yejH | | ygfB | | ygjE | | yibD | | yjbB | | yohC | | ytjB | |
| yecH | | yejK | | ygfI | | ygjF | | yibK | | yjbC | | yohD | | ytjC | |
| yecM | | yejL | | ygfX | | yhbH | | yibN | | yjbD | | yohF | | zapA | |
| yecN | | yejM | | ygfY | | yhbJ | | yibQ | | yjbJ | | yojI | | zntA | |
| yecO | | yfaD | | ygfZ | | yhcB | | yicE | | yjbN | | yojL | | zntR | |
| yecP | | yfaL | | yggE | | yhcM | | yicG | | yjbQ | | yqfA | | Z_RS00780 | |
| yegT | | yfaY | | yggG | | yhcO | | yicH | | yjbR | | yqfB | | Z_RS25620 | |
| yegU | | yfaZ | | yggJ | | yhdN | | yicI | | yjcB | | yqgB | | zupT | |
| yegV | | yfbB | | yggM | | yhdP | | yicJ | | yjcC | | yqgC | | zur | |
| yegW | | yfbQ | | yggN | | yheT | | yidA | | yjfC | | yqgD | |  | |
