# Supplementary material for: Stratified reconstruction of ancestral Escherichia coli diversification
Source: BMC Genomics. 2019 Dec 5;20:936. doi: 10.1186/s12864-019-6346-1 (PMC6896753; doi:10.1186/s12864-019-6346-1)
Supplement: Supplementary file 5 — Additional file 5: Figure S3. Ancestor phylogenetic reconstruction of E. coli phylogroups. (PPTX 77 kb) [file 12864_2019_6346_MOESM5_ESM.pptx]

## Slide 1
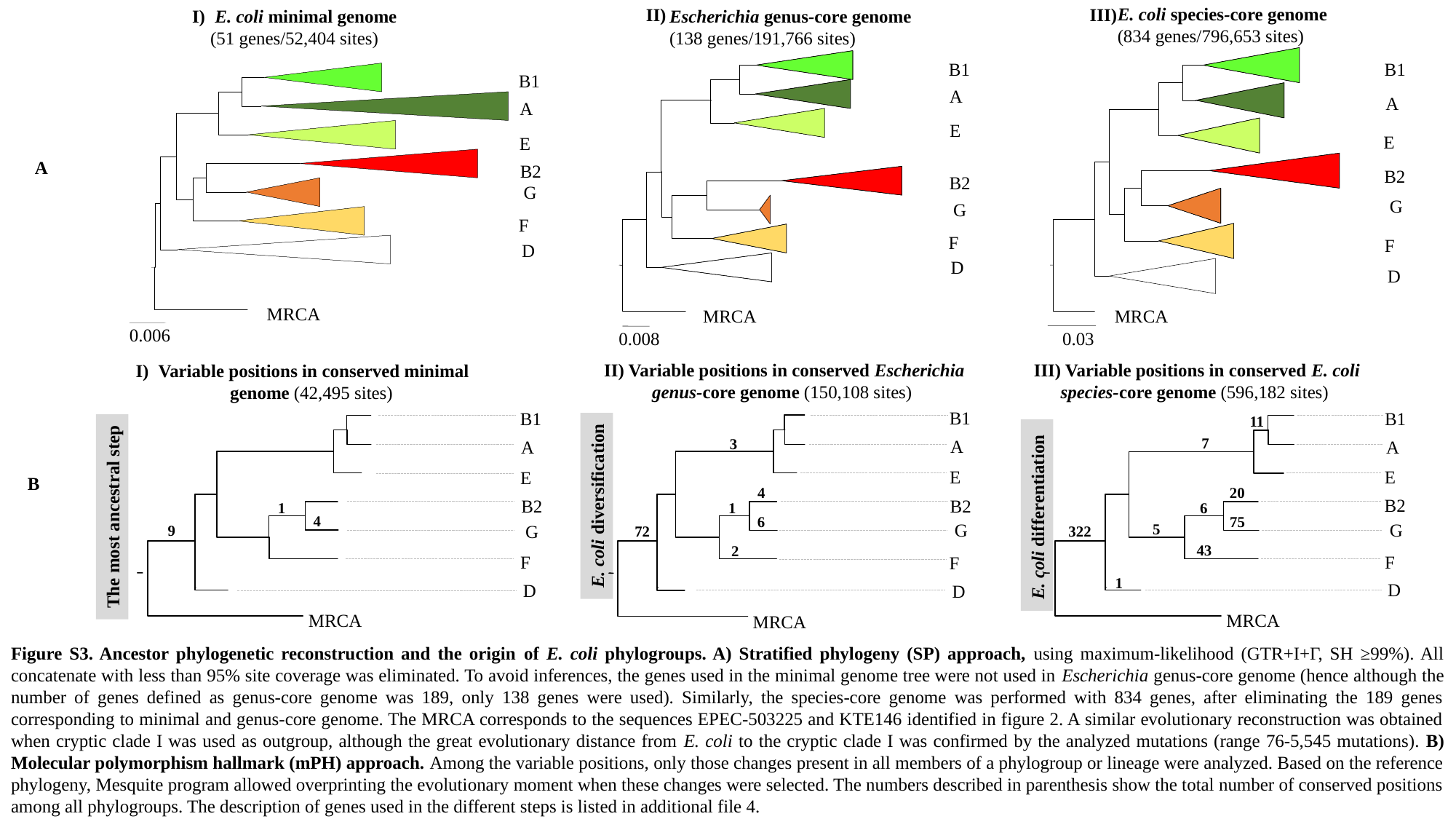

E. coli species-core genome
(834 genes/796,653 sites)
II)
III)
I)
 E. coli minimal genome
(51 genes/52,404 sites)
Escherichia genus-core genome
(138 genes/191,766 sites)
0.03
0.008
B1
A
G
F
D
MRCA
B1
A
G
F
D
0.006
B1
A
G
F
D
E
E
E
A
B2
B2
B2
MRCA
MRCA
III) Variable positions in conserved E. coli species-core genome (596,182 sites)
II) Variable positions in conserved Escherichia genus-core genome (150,108 sites)
Variable positions in conserved minimal genome (42,495 sites)
B1
B1
A
G
F
D
B1
11
3
4
1
6
72
2
MRCA
1
7
A
A
E
E
E
B
20
B2
B2
B2
E. coli diversification
6
E. coli differentiation
The most ancestral step
4
75
5
9
322
G
G
43
F
F
1
D
D
MRCA
MRCA
Figure S3. Ancestor phylogenetic reconstruction and the origin of E. coli phylogroups. A) Stratified phylogeny (SP) approach, using maximum-likelihood (GTR+I+Γ, SH ≥99%). All concatenate with less than 95% site coverage was eliminated. To avoid inferences, the genes used in the minimal genome tree were not used in Escherichia genus-core genome (hence although the number of genes defined as genus-core genome was 189, only 138 genes were used). Similarly, the species-core genome was performed with 834 genes, after eliminating the 189 genes corresponding to minimal and genus-core genome. The MRCA corresponds to the sequences EPEC-503225 and KTE146 identified in figure 2. A similar evolutionary reconstruction was obtained when cryptic clade I was used as outgroup, although the great evolutionary distance from E. coli to the cryptic clade I was confirmed by the analyzed mutations (range 76-5,545 mutations). B) Molecular polymorphism hallmark (mPH) approach. Among the variable positions, only those changes present in all members of a phylogroup or lineage were analyzed. Based on the reference phylogeny, Mesquite program allowed overprinting the evolutionary moment when these changes were selected. The numbers described in parenthesis show the total number of conserved positions among all phylogroups. The description of genes used in the different steps is listed in additional file 4.
