# Supplementary material for: Stratified reconstruction of ancestral Escherichia coli diversification
Source: BMC Genomics. 2019 Dec 5;20:936. doi: 10.1186/s12864-019-6346-1 (PMC6896753; doi:10.1186/s12864-019-6346-1)
Supplement: Supplementary file 6 — Additional file 6: Figure S4. Chromosomal size of E. coli phylogroups. (PPTX 44 kb) [file 12864_2019_6346_MOESM6_ESM.pptx]

## Slide 1
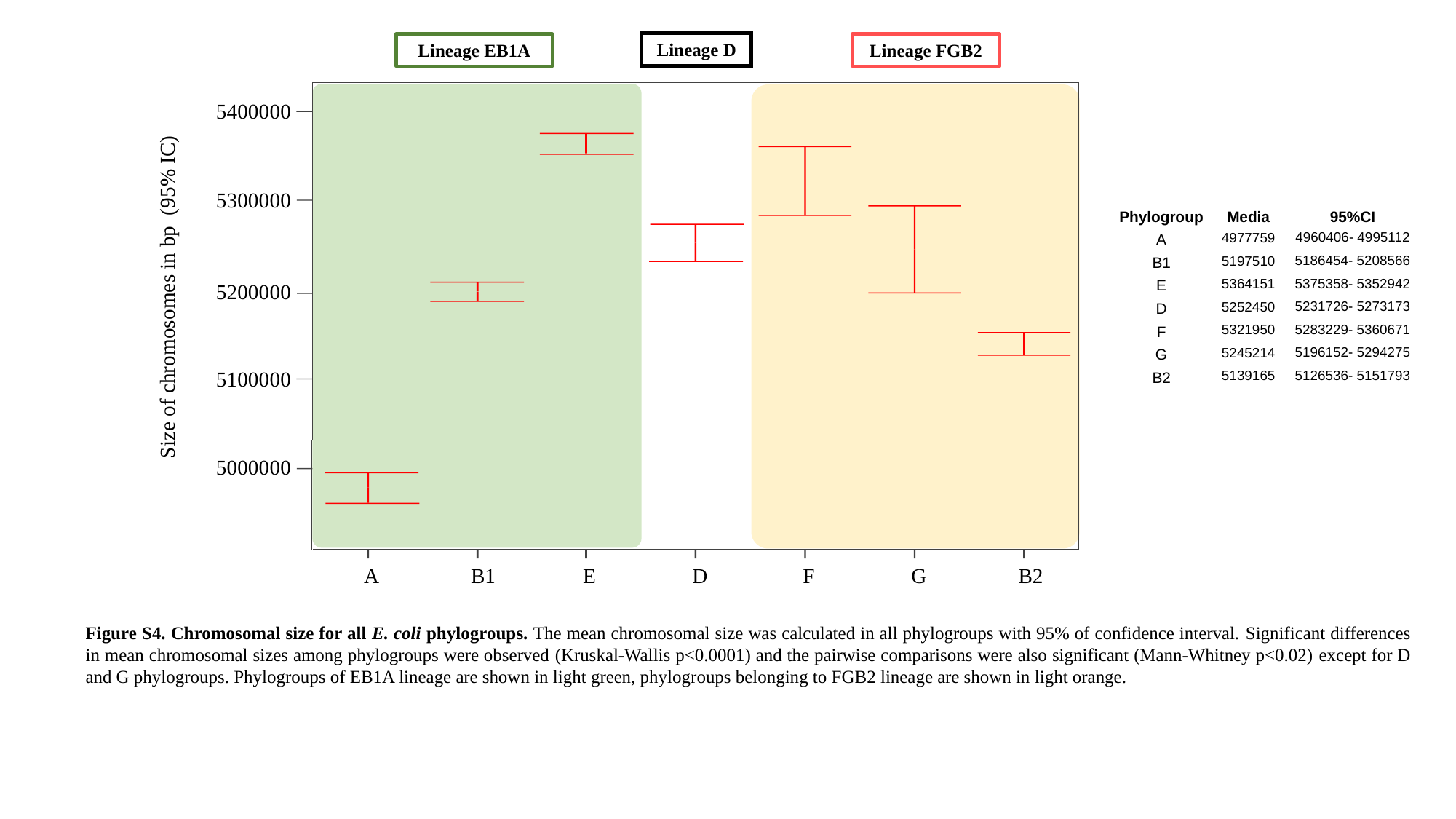

5400000
5300000
5200000
5100000
5000000
A
B1
E
D
F
G
B2
Size of chromosomes in bp (95% IC)
Lineage D
Lineage EB1A
Lineage FGB2
| Phylogroup | Media | 95%CI |
| --- | --- | --- |
| A | 4977759 | 4960406- 4995112 |
| B1 | 5197510 | 5186454- 5208566 |
| E | 5364151 | 5375358- 5352942 |
| D | 5252450 | 5231726- 5273173 |
| F | 5321950 | 5283229- 5360671 |
| G | 5245214 | 5196152- 5294275 |
| B2 | 5139165 | 5126536- 5151793 |
Figure S4. Chromosomal size for all E. coli phylogroups. The mean chromosomal size was calculated in all phylogroups with 95% of confidence interval. Significant differences in mean chromosomal sizes among phylogroups were observed (Kruskal-Wallis p<0.0001) and the pairwise comparisons were also significant (Mann-Whitney p<0.02) except for D and G phylogroups. Phylogroups of EB1A lineage are shown in light green, phylogroups belonging to FGB2 lineage are shown in light orange.
