# Supplementary material for: Stratified reconstruction of ancestral Escherichia coli diversification
Source: BMC Genomics. 2019 Dec 5;20:936. doi: 10.1186/s12864-019-6346-1 (PMC6896753; doi:10.1186/s12864-019-6346-1)
Supplement: Supplementary file 7 — Additional file 7: Figure S5. Inferred frequencies of accumulated mutations in the E. coli branches. (PPTX 49 kb) [file 12864_2019_6346_MOESM7_ESM.pptx]

## Slide 1
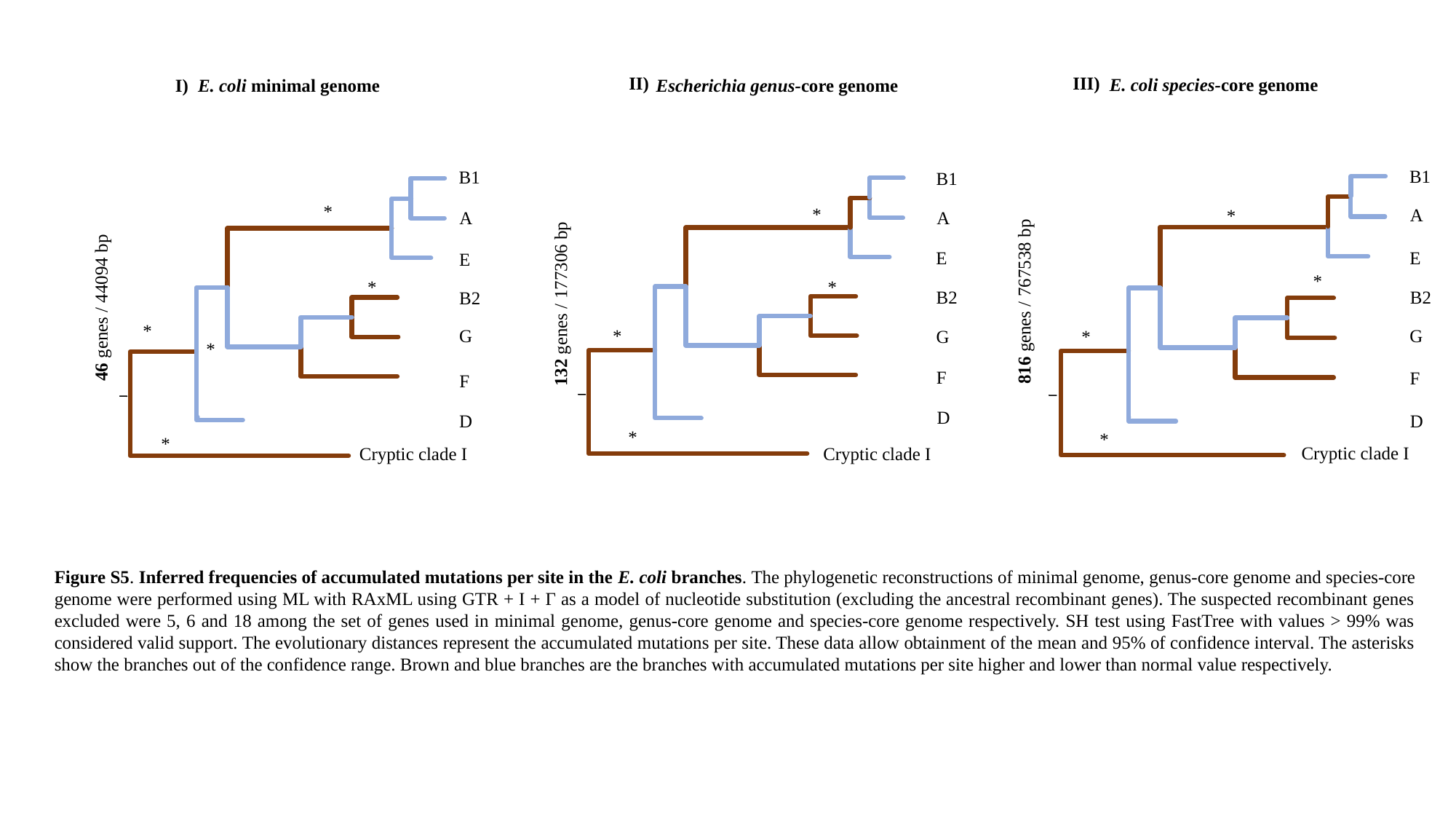

II)
III)
E. coli species-core genome
 E. coli minimal genome
Escherichia genus-core genome
I)
B1
A
E
B2
G
F
D
Cryptic clade I
B1
A
E
B2
G
F
D
Cryptic clade I
B1
A
E
B2
G
F
D
Cryptic clade I
*
*
*
46 genes / 44094 bp
816 genes / 767538 bp
*
132 genes / 177306 bp
*
*
*
*
*
*
*
*
*
Figure S5. Inferred frequencies of accumulated mutations per site in the E. coli branches. The phylogenetic reconstructions of minimal genome, genus-core genome and species-core genome were performed using ML with RAxML using GTR + I + Γ as a model of nucleotide substitution (excluding the ancestral recombinant genes). The suspected recombinant genes excluded were 5, 6 and 18 among the set of genes used in minimal genome, genus-core genome and species-core genome respectively. SH test using FastTree with values > 99% was considered valid support. The evolutionary distances represent the accumulated mutations per site. These data allow obtainment of the mean and 95% of confidence interval. The asterisks show the branches out of the confidence range. Brown and blue branches are the branches with accumulated mutations per site higher and lower than normal value respectively.
