# Supplementary material for: Stratified reconstruction of ancestral Escherichia coli diversification
Source: BMC Genomics. 2019 Dec 5;20:936. doi: 10.1186/s12864-019-6346-1 (PMC6896753; doi:10.1186/s12864-019-6346-1)
Supplement: Supplementary file 8 — Additional file 8: Figure S6. Ancestral recombination between E. coli phylogroups. (PPTX 59 kb) [file 12864_2019_6346_MOESM8_ESM.pptx]

## Slide 1
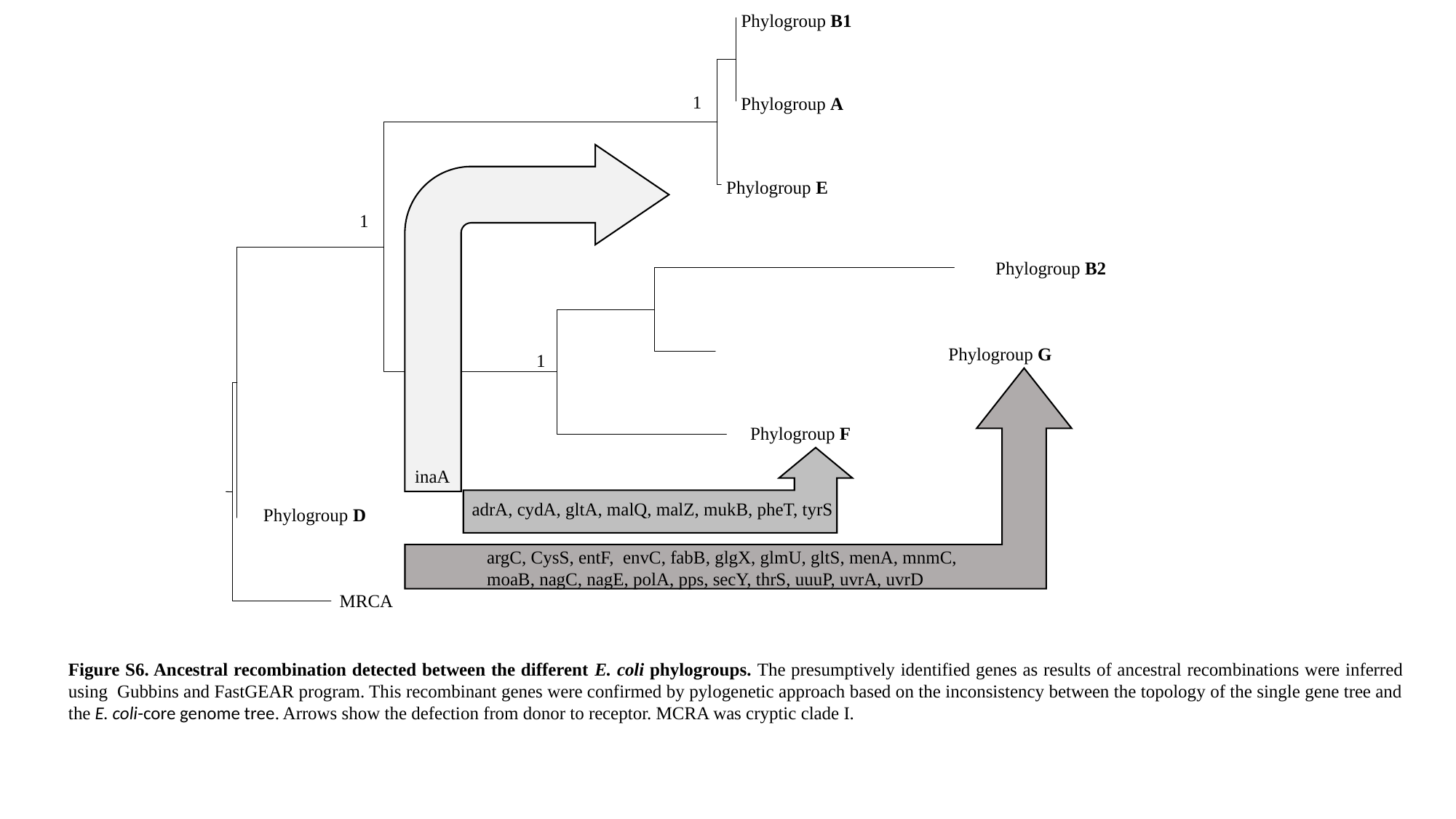

Phylogroup B1
1
Phylogroup A
Phylogroup E
1
Phylogroup B2
Phylogroup G
1
Phylogroup F
Phylogroup D
inaA
adrA, cydA, gltA, malQ, malZ, mukB, pheT, tyrS
argC, CysS, entF, envC, fabB, glgX, glmU, gltS, menA, mnmC,
moaB, nagC, nagE, polA, pps, secY, thrS, uuuP, uvrA, uvrD
MRCA
Figure S6. Ancestral recombination detected between the different E. coli phylogroups. The presumptively identified genes as results of ancestral recombinations were inferred using Gubbins and FastGEAR program. This recombinant genes were confirmed by pylogenetic approach based on the inconsistency between the topology of the single gene tree and the E. coli-core genome tree. Arrows show the defection from donor to receptor. MCRA was cryptic clade I.
