# Supplementary material for: Stratified reconstruction of ancestral Escherichia coli diversification
Source: BMC Genomics. 2019 Dec 5;20:936. doi: 10.1186/s12864-019-6346-1 (PMC6896753; doi:10.1186/s12864-019-6346-1)
Supplement: Supplementary file 9 — Additional file 9: Table S5. Ancestral genes gain and loss by E. coli phylogroups. [file 12864_2019_6346_MOESM9_ESM.docx]

**Table S5** Genes segregated during early stages of evolution of *Escherichia coli* phylogroups

| **Lineage** | **Gained genes** | **COG Categ** | **Lost genes** | **COG Categ** |
| --- | --- | --- | --- | --- |
| **EB1A** | ECNA114_RS09110  ECO26_3325  ECO26_3326  etp  xylE  ybdR  yccC  yccZ  yecT  yfgJ  yiaT  yiaU  yiaV  yiaW  ymcA  ymcB  ymcC  ymcD  ynjI  yraH  yraI  yraJ  yraK  Z_RS03315 | S  M  M  M  G  R  R  M  S  S  M  M  M  M  M  M  M  M  S  M/O  M/O  M/O  M/O  R | ECNA114_RS14560  ECNA114_RS21110  ECSMS35_RS01565  ECSMS35_RS01640  ECSMS35_RS01645  ECSMS35_RS08230  ECSMS35_RS08795  ECSMS35_RS10365  ECSMS35_RS10370  ECSMS35_RS11215  ECSMS35_RS13015  ECSMS35_RS13450  ECSMS35_RS13455  ECSMS35_RS13460  ECSMS35_RS13465  ECSMS35_RS14455  ECSMS35_RS14725  ECSMS35_RS15515  ECSMS35_RS16005  ECSMS35_RS16010  ECSMS35_RS16840  ECSMS35_RS16845  ECSMS35_RS16850  ECSMS35_RS19835  ECSMS35_RS19840  ECSMS35_RS20240  ECSMS35_RS20245  ECSMS35_RS20250  ECSMS35_RS20255  ECSMS35_RS20260  ECSMS35_RS20265  ECSMS35_RS20270  ECSMS35_RS20480  ECSMS35_RS21925  ECSMS35_RS22090  ECUMN_1481  ECUMN_4186  G840_RS11445 | S  S  S  R/K  R  S  X  R/K  S  X  X  S  R/V  R/V  S  V/U  X  R  R  X  R  R  O  S  S  O  S  V  S  V  V  V/R  S  X  R  S  M  N |

| **Sublineage** | **Gained genes** | **COG Categ** | **Lost genes** | **COG Categ** |
| --- | --- | --- | --- | --- |
| **B1A** | bglB  bglF  bglG  bglH  cbrB  ECO26_1856  ECSMS35_RS23515  ECSMS35_RS23520  ECSMS35_RS23525  ECSMS35_RS23530  ECSMS35_RS23535  ECSMS35_RS23540  mngA2  mngB  setA  ycaK  yfmA  yieK  yieL | G  G  G  G  K  S  C  R/I  R/I  G  R/I  R/K  G/V  G/V  G/R  R/V  S  R/G  R/G | ECNA114_RS00190  ECNA114_RS00195  ECSMS35_RS00380  ECSMS35_RS01630  ECSMS35_RS01635  ECSMS35_RS01650  ECSMS35_RS02170  ECSMS35_RS02640  ECSMS35_RS02645  ECSMS35_RS02650  ECSMS35_RS02655  ECSMS35_RS03675  ECSMS35_RS03680  ECSMS35_RS03685  ECSMS35_RS03690  ECSMS35_RS03695  ECSMS35_RS03700  ECSMS35_RS03705  ECSMS35_RS03710  ECSMS35_RS03715  ECSMS35_RS03720  ECSMS35_RS03725  ECSMS35_RS03730  ECSMS35_RS03735  ECSMS35_RS04285  ECSMS35_RS04290  ECSMS35_RS04455  ECSMS35_RS04460  ECSMS35_RS04465  ECSMS35_RS09855  ECSMS35_RS09860  ECSMS35_RS09865  ECSMS35_RS09870  ECSMS35_RS09875  ECSMS35_RS09880  ECSMS35_RS12140  ECSMS35_RS12625  ECSMS35_RS14140  ECSMS35_RS14145  ECSMS35_RS15720  ECSMS35_RS19185  ECSMS35_RS19190  ECSMS35_RS19195  ECSMS35_RS19200  ECSMS35_RS19205  ECSMS35_RS19210  ECSMS35_RS19215  ECSMS35_RS19220  ECSMS35_RS19735  ECSMS35_RS20280  ECSMS35_RS20285  ECSMS35_RS20290  ECSMS35_RS20330  ECSMS35_RS20335  ECSMS35_RS20585  ECSMS35_RS20590  ECSMS35_RS20595  ECSMS35_RS20600  ECSMS35_RS20605  ECSMS35_RS20610  ECSMS35_RS21625  ECUMN_0020  ECUMN_0021  ECUMN_0457  hmuV  siiBA  siiCA  siiEA  Z_RS23075 | X/V/R  X/V/R  U  T  R/K  Q  S  S  S  S  S  R/K  C  C  C  S  S  E  C/E  C/E  C/E  S  S  S  K  V  S  S  S  P  P  P  K  P  P  G/R  S/M  K/E  K/S  G/R  P  P  S  P  P  P  S  P  R/K/D  R/V  R/V  K  S  S  D  S  S  S  S  S  G/R  M  M  S  P  U  U  U  S |

| **Phylogroup** | **Gained genes** | **COG Categ** | **Lost genes** | **COG Categ** |
| --- | --- | --- | --- | --- |
| **E** | ECNA114_RS01545  ECNA114_RS01550  ECNA114_RS01555  ECNA114_RS21230  ECSMS35_RS09280  ECSMS35_RS09285  ECSMS35_RS09290  ECSMS35_RS09295  ECSMS35_RS09300  ECSMS35_RS11060  ECSMS35_RS12650  ECSMS35_RS16765  ECSMS35_RS16770  ECSMS35_RS16775  ECSMS35_RS16780  ECSMS35_RS16785  ECSMS35_RS16790  ECSMS35_RS18720  ECSMS35_RS18770  ECSMS35_RS18775  ECSMS35_RS24285  G840_RS21410  Z_RS02030  Z_RS04570 | U  U  K  R  R/V  R/M/V  V  R/U  R/K  N  M  R/K  R/P  R/P  R/P  R/P  P  S  L  L  S  L  R  U | dgoA  dgoK  dgoR  ECNA114_RS01560  ECNA114_RS23675  ECO26_0395  ECO26_4352  ECO26_4592  ECO26_4593  ECO26_4699  ECO26_4700  ECO26_4701  ECO26_4850  ECO26_4852  ECO26_5203  ECSMS35_RS05530  ECSMS35_RS12030  ECSMS35_RS12035  ECSMS35_RS12040  ECSMS35_RS12045  ECSMS35_RS12050  ECSMS35_RS12055  ECSMS35_RS12945  ECSMS35_RS20460  ECUMN_0881  ECUMN_4100  lyx  sgbE  sgbH  sgbU  xapA  xapB  xapR  yadN  yaiO  yaiP  yaiS  ybdO  ybgO  ybiA  ycfZ  yfeN  ygbI  ygbJ  ygbK  ygbL  ygbM  ygbN  yiaK  yiaL  yiaM  yiaN  yiaO | G  G  G  S  R  R  S  K  R  R/K  S/V  R  M  N  C  G  K  K/I  I  I  I  I  R  R/M/G  S  S  G  G  G  G  F  F  F  R/O/N  G  G  G  R/K  R/O/N  N  S  R/G  K  R  S  R  S  S  G  G  G  G  G |

| **Phylogroup** | **Gained genes** | **COG Categ** | **Lost genes** | **COG Categ** |
| --- | --- | --- | --- | --- |
| **A** | ECNA114_RS17540  ECNA114_RS17545  ECNA114_RS17550  ECNA114_RS17555  ECNA114_RS17560  ECNA114_RS17565  ECNA114_RS17570  ECNA114_RS17575  ECNA114_RS17580  ECNA114_RS17585  ECNA114_RS17590  ECNA114_RS17595  ECNA114_RS17600  ECNA114_RS17605  ECNA114_RS17615  ECNA114_RS17620  ECSMS35_RS05370  ECSMS35_RS05375  ECSMS35_RS19545  ECSMS35_RS19550  ECSMS35_RS22870  ECSMS35_RS22875  ECSMS35_RS22880  ECSMS35_RS22885  ECSMS35_RS22890  ECSMS35_RS22895 | U  U  U  U  U  U  U  U  U  U  U  U  U  U  R/P  R/C  X  X  X  X  R/G  G  G  G  R/G  G | ECO26_0359  ECO26_0360  ECO26_0361  ECO26_0362  ECO26_3806  ECO26_3807  ECO26_3808  ECO26_3809  ECO26_4424  ECO26_4426  ECO26_4699  ECO26_4700  ECO26_4701  ECO26_4851  ECSMS35_RS21080  ECSMS35_RS25865  lfpB | S  G  G  R  S  R/V  R/V  R/K  U  R/P  R/K  S/V  R  O  R/Q  S  O |
| **B1** | ECO26_4197  ECO26_4198  ECO26_4199  ECO26_4200  ECO26_4514  ECO26_4515  ECO26_4516  ECO26_4699  ECO26_4700  ECO26_4701  ECSMS35_RS00745  G840_RS02840 | M  M  M  M  S  S  S  R/K  S/V  R  R/O/N  E/G/Q | ECSMS35_RS09450  ECSMS35_RS21530 | S  S |

| **Lineage** | **Gained genes** | **COG Categ.** | **Lost genes** | **COG Categ.** |
| --- | --- | --- | --- | --- |
| **FGB2** | ECNA114_RS02350  ECO26_3884  ECO26_3885  ECO26_3886  ECO26_3887  ECO26_3888  ECSMS35_RS00745  ECSMS35_RS09280  ECSMS35_RS09285  ECSMS35_RS09290  ECSMS35_RS09295  ECSMS35_RS09300  ECSMS35_RS09440  ECSMS35_RS09825  ECSMS35_RS16765  ECSMS35_RS16770  ECSMS35_RS16775  ECSMS35_RS16780  ECSMS35_RS16785  ECSMS35_RS16790  ECSMS35_RS20100  ECSMS35_RS20105  ECSMS35_RS20110  ECSMS35_RS20115  ECSMS35_RS20120  ECSMS35_RS20125  ECSMS35_RS20130  ECSMS35_RS20135  ECSMS35_RS21220  ECSMS35_RS21225  ECSMS35_RS21230  ECSMS35_RS21235  ECSMS35_RS21240  ECSMS35_RS21245  ECSMS35_RS22070  ECSMS35_RS22075  ECSMS35_RS22080  ECSMS35_RS22085  ECSMS35_RS22870  ECSMS35_RS22875  ECSMS35_RS22880  ECSMS35_RS22885  ECSMS35_RS22890  ECSMS35_RS22895  ECSMS35_RS23515  ECSMS35_RS23520  ECSMS35_RS23525  ECSMS35_RS23530  ECSMS35_RS23535  ECSMS35_RS23540  G840_RS02840  G840_RS22560  yfmA | V/R  R/H  R/M/G  R/E  R/G  R/K  R/O/N  R/V  R/M/V  V  R/U  R/K  S  M/R  R/K  R/P  R/P  R/P  R/P  P  G  G/R  C/G  C/G  G  G  G  G  P  P  P  G  G  R/K  F/V  F/V  F/V  S  R/G  G  G  G  R/G  G  C  R/I  R/I  G  R/I  R/K  E/G/Q  N  S | ECO26_3324  ECUMN_0457  htrC  yagQ  yagR  yagS  yagT  ybiA  ycgV  yjcF  Z_RS09225 | O  S  R/V  R/V  R/V  R/V  R/V  N  R/N  S  S |

| **Sublineage** | **Gained genes** | **COG Categ** | **Lost genes** | **COG Categ** |
| --- | --- | --- | --- | --- |
| **GB2** | bglB  bglF  bglG  bglH  cbrB  ECNA114_RS01545  ECNA114_RS01550  ECNA114_RS01555  ECNA114_RS02380  ECNA114_RS02385  ECNA114_RS07900  ECNA114_RS07905  ECNA114_RS07910  ECNA114_RS09110  ECNA114_RS14750  ECNA114_RS14755  ECNA114_RS14760  ECNA114_RS14765  ECNA114_RS14770  ECNA114_RS14775  ECNA114_RS14780  ECNA114_RS14785  ECNA114_RS14790  ECNA114_RS14810  ECNA114_RS14815  ECNA114_RS14825  ECNA114_RS14830  ECNA114_RS14835  ECNA114_RS14840  ECNA114_RS14845  ECNA114_RS14850  ECNA114_RS14855  ECNA114_RS17540  ECNA114_RS17545  ECNA114_RS17550  ECNA114_RS17555  ECNA114_RS17560  ECNA114_RS17565  ECNA114_RS17570  ECNA114_RS17575  ECNA114_RS17580  ECNA114_RS17585  ECNA114_RS17590  ECNA114_RS17595  ECNA114_RS17600  ECNA114_RS17605  ECNA114_RS17615  ECNA114_RS17620  ECNA114_RS18625  ECNA114_RS19660  ECNA114_RS19665  ECNA114_RS22855  ECNA114_RS22860  ECO26_4514  ECO26_4515  ECO26_4516  G840_RS05630  G840_RS05635  G840_RS05640  G840_RS05645  G840_RS05650  G840_RS05655  G840_RS05660  G840_RS21410  yieK  yieL | G  G  G  G  K  U  U  K  S  S  G  U  U  S  U  U  U  U  R  U  U  U  U  U  S  U  U  U  U  U  U  U  U  U  U  U  U  U  U  U  U  U  U  U  U  U  R/P  R/C  S  R  S  G  V  S  S  S  M  M  M  M  M  O  M  L  R/G  R/G | chpA  chpB  chpR  chpS  ECO26_0022  ECO26_1556  ECO26_2069  ECO26_3231  ECO26_4352  ECO26_4424  ECO26_4426  ECSMS35_RS00380  ECSMS35_RS02170  ECSMS35_RS20240  ECSMS35_RS20245  ECSMS35_RS20250  ECSMS35_RS20255  ECSMS35_RS20260  ECSMS35_RS20265  ECSMS35_RS20270  ECSMS35_RS20330  ECSMS35_RS20335  ECSMS35_RS20585  ECSMS35_RS20590  ECSMS35_RS20595  ECSMS35_RS20600  ECSMS35_RS20605  ECSMS35_RS20610  ECUMN_1481  ECUMN_2981  ECUMN_4186  glvC  ycbF  ycbQ  ycbR  ycbS  ycbT  ycbU  ycbV  yidI | V  V  V  V  U  S  U  S  S  S  R/P  U  S  O  S  V  S  V  V  V/R  S  S  D  S  S  S  S  S  S  S  M  G  O  O  O  O  O  O  O  R |

| **Phylogroup** | **Gained genes** | | | **COG Categ** | **Lost genes** | | **COG Categ** |
| --- | --- | --- | --- | --- | --- | --- | --- |
| **F** | dinJ  ECO26_4197  ECO26_4198  ECO26_4199  ECO26_4200  ECSMS35_RS00090  ECSMS35_RS01180  ECSMS35_RS03530  ECSMS35_RS05370  ECSMS35_RS05375  ECSMS35_RS10085  ECSMS35_RS11060  ECSMS35_RS12615  ECSMS35_RS12620  ECSMS35_RS12645  ECSMS35_RS12650  ECSMS35_RS12655  ECSMS35_RS13455  ECSMS35_RS18595  ECSMS35_RS18600  ECSMS35_RS18720  ECSMS35_RS18725  ECSMS35_RS18770  ECSMS35_RS18775  ECSMS35_RS19545  ECSMS35_RS19550  ECSMS35_RS22505 | | | V  M  M  M  M  P  R  S  X  X  S  N  R  L  M  M  M  R/V  V  K  S  R  L  L  X  X  K/E | ECNA114_RS21590  ECNA114_RS25250  ECO26_3422  ECO26_3423  ECO26_3424  G840_RS0124460  G840_RS10745  ybgD  ybgQ | | S  R/G  G  G  G  S  V/R  M  M |
| **G** | ECNA114_RS20565  ECNA114_RS20570  ECNA114_RS20575  ECNA114_RS20580  ECNA114_RS20585  ECNA114_RS20590  ECNA114_RS20595  ECNA114_RS20600  ECSMS35_RS03530  ECSMS35_RS09435  ECSMS35_RS10085  ECSMS35_RS18595  ECSMS35_RS18600  etp  G840_RS0124605  G840_RS0124965  G840_RS05520  G840_RS05525  G840_RS08920  G840_RS11440  G840_RS11450  G840_RS11455  G840_RS15825  G840_RS19720  G840_RS22575  G840_RS22580  G840_RS22585  yccC  yccZ  yiaT  yiaU  yiaV  yiaW  yjeO  ymcA  ymcB  ymcC  ymcD | | | G  G  G  G  G  G  R  G  S  S  S  V  K  M  S  S  V  V  V  N  M  M  V  V  M  M  M  R  M  M  M  M  M  S  M  M  M  M | ECSMS35_RS19735  setC  ydeJ  ECSMS35_RS20290 | | R/K/D  G  S  K |
| **B2** | | ECNA114_RS21230  ECNA114_RS21830  ECNA114_RS21835  ECNA114_RS21840  ECNA114_RS21845  ECNA114_RS21850  ECNA114_RS21855  ECNA114_RS21860  ECNA114_RS21865  ECSMS35_RS00090  ECSMS35_RS12645  ECSMS35_RS12655 | R  G  G  G  G  P  G  K  R  P  M  M | | abgA  abgB  abgR  argK  arsB  arsR  cynR  cynS  cynT  cynX  dcuD  ddpA  ddpB  ddpC  ddpD  ddpX  ECO26_3806  ECO26_3807  ECO26_3808  ECO26_3809  ECO26_4592  ECO26_4593  ECO26_4810  ECO26_4850  ECO26_4851  ECO26_4852  ECO26_5171  ECSMS35_RS04285  ECSMS35_RS04290  ECSMS35_RS07395  ECSMS35_RS08370  ECSMS35_RS08400  ECSMS35_RS08770  ECSMS35_RS09910  ECSMS35_RS13340  ECSMS35_RS14045  ECSMS35_RS14050  ECSMS35_RS14065  ECSMS35_RS19075  ECSMS35_RS20360  ECSMS35_RS20640  ECSMS35_RS22635  ECUMN_0018  ECUMN_0019  ECUMN_3079  ECUMN_4100  envY  fimZ  focB  G840_RS0124865  G840_RS11445  glvG  hcaB  hcaC  hcaD  hcaE  hcaF  hcaR  hyfA  hyfB  hyfC  hyfD  hyfE  hyfF  hyfG  hyfH  hyfJ  lfpB  lsrA  lsrB  lsrC  lsrD  lsrR  melB  puuA  puuB  puuC  puuD  puuR  scpB  sfmA  sfmC  sfmD  sfmF  sfmH  siiBA  siiCA  siiEA  tisB  yahL  ybgD  ybgQ  ybiU  ycaM  ycaN  ydbD  yddL  ydeK  yeaV  yeaW  yeaX  yfdF  yfeT  yhaI  yhiJ  yhiM  yiaM  yidX  yncB  Z_RS18500 | Q/V  Q/V  Q/V  Q/V  Q/V  Q/V  Q/V  Q/V  Q/V  Q/V  R  E  E  E  E  E  S  R/V  R/V  R/K  K  R  S  M  O  N  S  K  V  S  G  R  R  R  C  S  S  G  S  S  M  U  R/M  R/M  S  S  R  K  R/G  S  N  R/G  Q  Q  Q  Q  Q  Q  Q  Q  Q  Q  Q  Q  Q  Q  Q  O  R  R  R  R  R  G  Q  Q  Q  Q  Q  Q  O  O  O  O  O  U  U  U  V  S  M  M  S  R  K  Q/V  S  S  R  R  R  S  K  S  S  R/V  R  S  Q  L | |

**Table S5.** Reference sequences where to find the genes identified by their locus_tag

| **Locus_tag** | **Reference sequences** |
| --- | --- |
| ECNA114_  ECO26_  ECSMS35_  ECUMN_  G840_  Z_ | NC_017644  NC_013361  NC_010498  CU928163  NZ_KE700731  NC_002655 |
