# Supplementary material for: Stratified reconstruction of ancestral Escherichia coli diversification
Source: BMC Genomics. 2019 Dec 5;20:936. doi: 10.1186/s12864-019-6346-1 (PMC6896753; doi:10.1186/s12864-019-6346-1)
Supplement: Supplementary file 10 — Additional file 10: Figure S7. E. coli phylogroup I phylogeny. (PPTX 119 kb) [file 12864_2019_6346_MOESM10_ESM.pptx]

## Slide 1
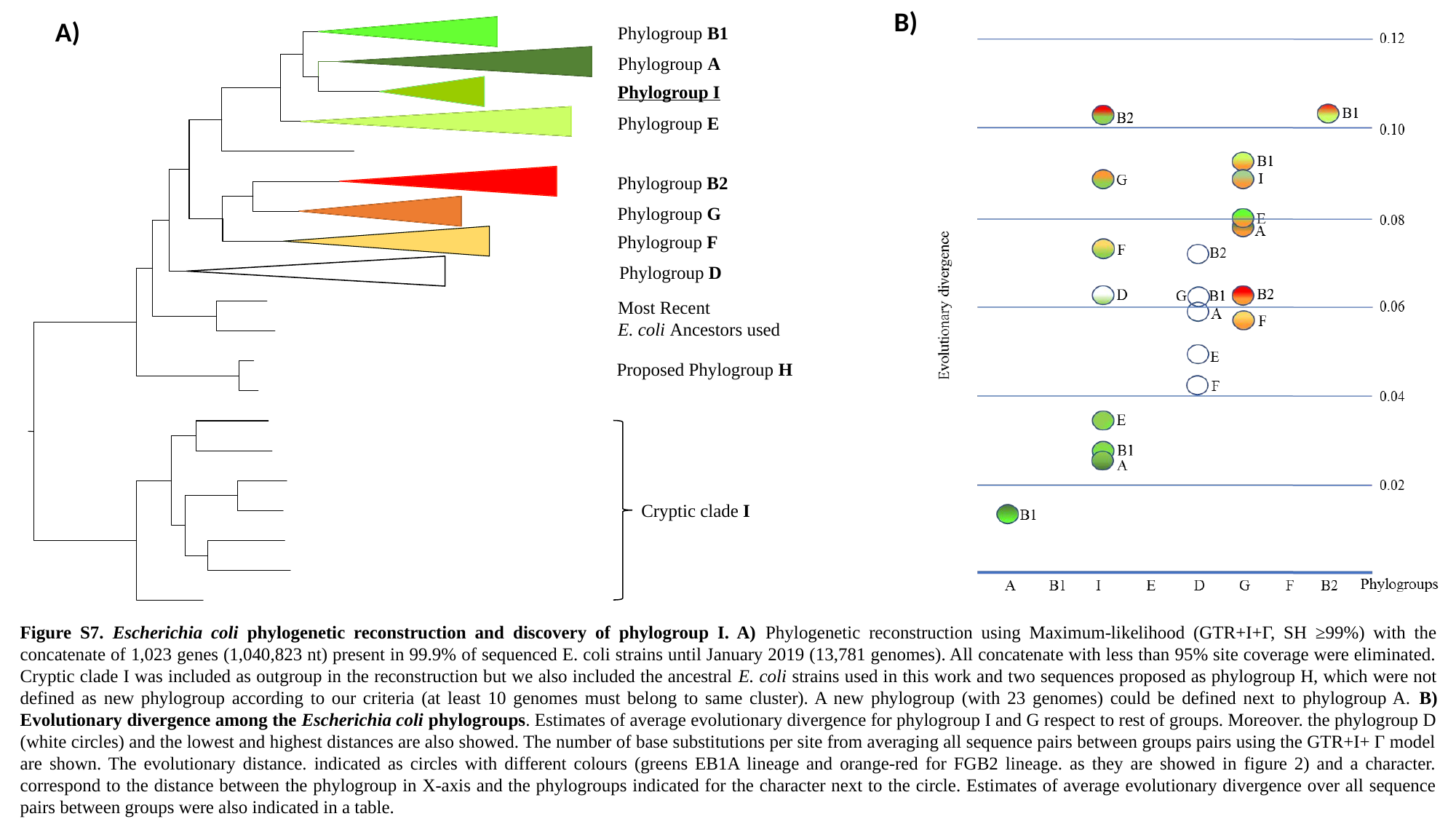

B)
A)
Phylogroup B1
Phylogroup A
Phylogroup I
Phylogroup E
Phylogroup B2
Phylogroup G
Phylogroup F
Phylogroup D
Most Recent
E. coli Ancestors used
Proposed Phylogroup H
Cryptic clade I
Figure S7. Escherichia coli phylogenetic reconstruction and discovery of phylogroup I. A) Phylogenetic reconstruction using Maximum-likelihood (GTR+I+Γ, SH ≥99%) with the concatenate of 1,023 genes (1,040,823 nt) present in 99.9% of sequenced E. coli strains until January 2019 (13,781 genomes). All concatenate with less than 95% site coverage were eliminated. Cryptic clade I was included as outgroup in the reconstruction but we also included the ancestral E. coli strains used in this work and two sequences proposed as phylogroup H, which were not defined as new phylogroup according to our criteria (at least 10 genomes must belong to same cluster). A new phylogroup (with 23 genomes) could be defined next to phylogroup A. B) Evolutionary divergence among the Escherichia coli phylogroups. Estimates of average evolutionary divergence for phylogroup I and G respect to rest of groups. Moreover. the phylogroup D (white circles) and the lowest and highest distances are also showed. The number of base substitutions per site from averaging all sequence pairs between groups pairs using the GTR+I+ Γ model are shown. The evolutionary distance. indicated as circles with different colours (greens EB1A lineage and orange-red for FGB2 lineage. as they are showed in figure 2) and a character. correspond to the distance between the phylogroup in X-axis and the phylogroups indicated for the character next to the circle. Estimates of average evolutionary divergence over all sequence pairs between groups were also indicated in a table.
