# Supplementary material for: Stratified reconstruction of ancestral Escherichia coli diversification
Source: BMC Genomics. 2019 Dec 5;20:936. doi: 10.1186/s12864-019-6346-1 (PMC6896753; doi:10.1186/s12864-019-6346-1)
Supplement: Supplementary file 11 — Additional file 11: Table S6. Genomes belonging to phylogroup I. [file 12864_2019_6346_MOESM11_ESM.docx]

**Table S6**. List of genomes allocated as phylogroup I

| **Accession number** | **Host** | **Country** | **Collection date** |
| --- | --- | --- | --- |
| MRWA01000000 | cow | Israel | 2016 |
| NIYL01000000 |  | Egypt | 2016 |
| NLMP01000000 | Gallus gallus | USA: CO | 1978 |
| NLUJ01000000 | Bos taurus | USA: PA | 1970 |
| NMNN01000000 | Sus scrofa scrofa | USA: PA | 1975 |
| NTEN01000000 |  | USA: FL | 2015 |
| NTEP01000000 |  | USA: FL | 2015 |
| NTEX01000000 |  | USA: FL | 2015 |
| PIUR01000000 | Bos taurus | USA: FL | 2016 |
| PJHU01000000 | Homo sapiens | China | 2016 |
| PKKW01000000 | Bos taurus | USA: FL | 2014 |
| QDJW01000000 | Bos taurus | USA: FL | 2014 |
| QDJX01000000 | Bos taurus | USA: FL | 2014 |
| QDJY01000000 | Bos taurus | USA: FL | 2014 |
| UCZI01000000 |  |  |  |
| NTFF01000000 |  | USA: FL | 2015 |
| QDKF01000000 | Bos taurus | USA: FL | 2011 |
| AMUQ01000000 |  |  |  |
| JUBT01000000 |  | USA | 2012 |
| JUBV01000000 |  | USA | 2012 |
| JUCI01000000 |  | USA | 2012 |
| JWZA01000000 |  | USA | 2012 |
| LM997334 | Human | Norway | 2011 |
