# Supplementary material for: Stratified reconstruction of ancestral Escherichia coli diversification
Source: BMC Genomics. 2019 Dec 5;20:936. doi: 10.1186/s12864-019-6346-1 (PMC6896753; doi:10.1186/s12864-019-6346-1)
Supplement: Supplementary file 12 — Additional file 12: Figure S8. Estimation of E. coli core genome. (PPTX 96 kb) [file 12864_2019_6346_MOESM12_ESM.pptx]

## Slide 1
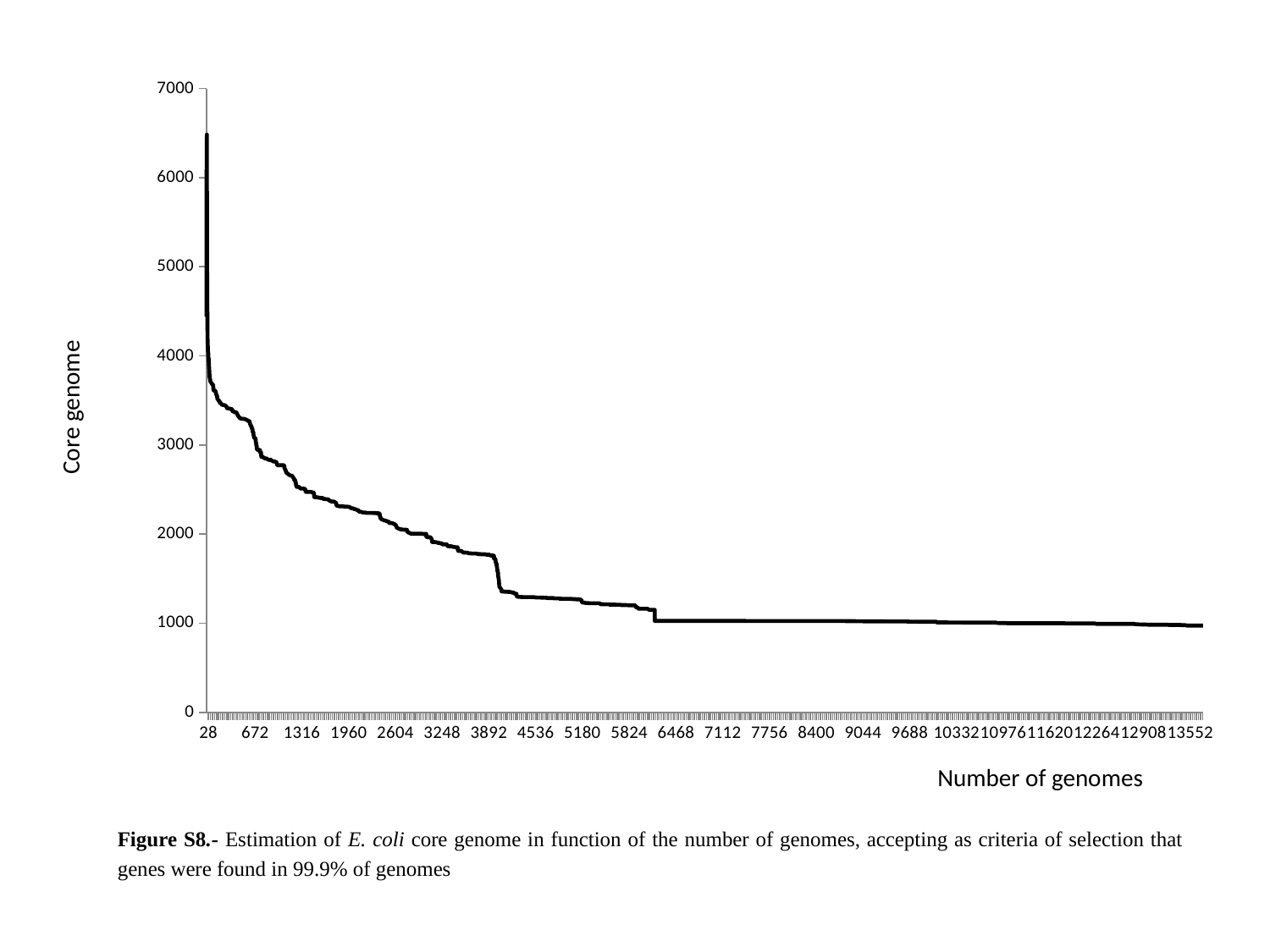

### Chart
| Category | |
|---|---|Core genome
Number of genomes
Figure S8.- Estimation of E. coli core genome in function of the number of genomes, accepting as criteria of selection that genes were found in 99.9% of genomes
